# Could It Be Snowing Microbes on Enceladus? Assessing Conditions in Its Plume and Implications for Future Missions

**DOI:** 10.1089/ast.2017.1665

**Published:** 2017-09-01

**Authors:** Carolyn C. Porco, Luke Dones, Colin Mitchell

**Affiliations:** ^1^ Space Science Institute, Boulder, Colorado.; ^2^ University of California, Berkeley, California.; ^3^ Southwest Research Institute, Boulder, Colorado.

## Abstract

We analyzed Cassini Imaging Science Subsystem (ISS) images of the plume of Enceladus to derive particle number densities for the purpose of comparing our results with those obtained from other Cassini instrument investigations. Initial discrepancies in the results from different instruments, as large as factors of 10–20, can be reduced to ∼2 to 3 by accounting for the different times and geometries at which measurements were taken. We estimate the average daily ice production rate, between 2006 and 2010, to be 29 ± 7 kg/s, and a solid-to-vapor ratio, S/V > 0.06. At 50 km altitude, the plume's peak optical depth during the same time period was *τ* ∼ 10^−3^; by 2015, it was ∼10^−4^. Our inferred differential size distribution at 50 km altitude has an exponent *q* = 3. We estimate the average geothermal flux into the sea beneath Enceladus' south polar terrain to be comparable to that of the average Atlantic, of order 0.1 W/m^2^. Should microbes be present on Enceladus, concentrations at hydrothermal vents on Enceladus could be comparable to those on Earth, ∼10^5^ cells/mL. We suggest the well-known process of bubble scrubbing as a means by which oceanic organic matter and microbes may be found in the plume in significantly enhanced concentrations: for the latter, as high as 10^7^ cells/mL, yielding as many as 10^3^ cells on a 0.04 m^2^ collector in a single 50 km altitude transect of the plume. Mission design can increase these numbers considerably. A lander mission, for example, catching falling plume particles on the same collector, could net, over 100 Enceladus days without bubble scrubbing, at least 10^5^ cells; and, if bubble scrubbing is at work, up to 10^8^ cells. Key Words: Enceladus—Microbe—Organic matter—Life detection. Astrobiology 17, 876–901.

## 1. Introduction

Cassini's exploration of the small saturnian moon Enceladus by both remote sensing and *in situ* investigations has demonstrated its status as a prime candidate for astrobiological study and the search for evidence of life. It is home to a sub-ice-shell global ocean (*e.g.,* Thomas *et al.,* 2016) of salty water (Postberg *et al.,* 2009), that is likely long-lived (*e.g.,* Fuller *et al.,* 2016; Lainey *et al.,* 2017) and vents through four long, prominent fractures in the south polar terrain (SPT) ice shell in fissure-type eruptions that take the form of more than 100 discrete geysers of vapor and icy particles, with faint sheets of material in between (Porco *et al.,* 2014; Teolis *et al.,* 2017, in this issue). The particles are accelerated in the conduits leading to the surface by outflowing vapor that is either exsolved and/or boiled from the liquid water column; speeds up to a few kilometers per second are not implausible (Teolis *et al.,* 2017, in this issue). The smaller the particles, the higher their velocities are.

The large plume formed by both discrete and continuous eruptions has been shown to vary in mass on a diurnal cycle (Hedman *et al.,* 2013) in response to tidal open/close forcing of the fractures from which the eruptions emerge (Nimmo *et al.,* 2014). The plume's contents—both vapor and solids—are largely water but contain small amounts of organic and other biologically significant compounds (*e.g.,* Waite *et al.,* 2009; Kopparla *et al.,* 2016; Postberg *et al.,* 2017). Recently, H_2_ has been detected in the vapor (Waite *et al.,* 2017), and evidence has also been found in the plume ice particles of seafloor hydrothermal activity with alkaline pH values and temperatures (Hsu *et al.,* 2015), similar to those of Lost City, a mid-ocean-ridge flanking, “low temperature,” hydrothermal zone in the mid-Atlantic.

Because Enceladus demonstrably possesses all the formal qualifications of an extraterrestrial habitable zone, there is already substantial activity underway in designing and planning future return missions that could address the more challenging questions that Cassini could not: Does the moon's plume contain evidence of biological activity? Might there even be microbes within the ice particles that form the visible plume, most of which snow back down onto the surface?

Answering these questions requires a mission design that allows collection of adequately sized samples and an instrument suite sufficiently advanced to analyze the contents of its plume in ways deeper and more precise than Cassini did. Various mission options exist in principle: Saturn orbiters, Enceladus orbiters, landers, and even sample returns. All involve, at some point in the mission, flights through the densest part of Enceladus' plume. Consequently, germane to the success of any mission are the questions of how much material is available for collection in a plume fly-through, how is it distributed in three dimensions, how does it vary in time, how rapidly does it accumulate on the surface, and what percentage of that material could be expected to be complex organic compounds or even microorganisms.

Over the course of Cassini's time at Saturn, there have been very few published measures of the size and spatial distributions of the ice particles in the plume. The photometric behavior of the plume observed by Cassini's Imaging Science Subsystem (ISS) in 2005 immediately indicated the presence of very small (roughly micron-sized) particles, diffracting light into high phase angles but sufficiently tenuous to be undetectable at phase angles below ∼130°, with most of the ejected material falling back down to the surface (*e.g.,* Porco *et al.,* 2006). A column mass abundance derived from the absolute brightness in the first high-resolution images of the plume taken in November 27, 2005, assuming an effective water ice particle radius of 1 μ and a broad size distribution, was ∼3 × 10^−3^ gm/m^2^ at ∼15 km above the surface at a latitude of ∼76°S (Porco *et al.,* 2006).

A Visual and Infrared Mapping Spectrometer (VIMS) set of remote measurements taken of the plume's near-IR spectrum and brightness in late 2005, also at high phase, resulted in estimates of the particle size distribution and volume number densities at several altitudes (Hedman *et al.,* 2009). Larger particles were present near the surface but absent at higher altitudes, consistent with the ISS result that most particles were falling down to the surface. VIMS estimated an eruption rate of anywhere from 2 to 200 kg/s in solids.

Early estimates of the total mass of the plume (Ingersoll and Ewald, 2011; henceforth IE11) from ISS images made use of a handful of very low-spatial-resolution, very high-phase images of the plume taken in 2006 to model the vertical profile of brightness in terms of mass flux leaving the surface. Estimates of particle volume number densities and variations with altitude were not reported. A mass production rate of ∼50 kg/s was derived, of which ∼9% was escaping Enceladus into Saturn's orbit. Another early estimate that made use of both CDA data and ISS images (Schmidt *et al.,* 2008) found a mass rate of ∼5 kg/s, with 0.5 kg/s escaping into the E ring.

*In situ* instruments like Cassini's Cosmic Dust Analyzer (CDA) and Radio and Plasma Wave Science (RPWS) experiment successfully sampled the plume particles November 2, 2009, during the 100 km E7 pass through the heart of the plume and directly over the south pole (Ye *et al.,* 2014). The peak particle densities and particle size distributions determined by each instrument were consistent with each other. A more recent analysis of the E7 flyby CDA data (Kempf *et al.,* 2016) found for a differential size distribution, *dN*/*dr* ∝ *r^-q^*, an exponent *q* ≈ 3.5 and a mass production rate of 2.5 kg/s, 20 times smaller than that derived by IE11 from ISS. An analysis of CDA data obtained during the closer, 50 km altitude, off-center E21 flyby on October 28, 2015, yielded, as expected, higher particle densities and a mass production rate of ∼5 kg/s, consistent with the earlier CDA result (Schmidt *et al.,* 2008) but a factor of 10 smaller than IE11.

Obviously for quantities of great interest, there are thus far, on the face of it, major discrepancies in the results from different Cassini instruments, with the remote sensing observations yielding higher masses and mass production rates in the solids than the *in situ* values. The plume's particle concentration is important for the design of future missions and estimating the amount of collected solid material and, from there, the expected abundance of biologically significant compounds. It is also necessary for determining the ratio of total mass of the plume in solids to the total mass in vapor, a quantity of critical value for understanding the eruption sources and mechanisms. Scientifically and programmatically, it is important that these results be reconciled.

Gao *et al.* (2016) attempted to explain these differences by proposing that the plume might consist of fractal aggregates. The aggregates have larger surface-to-mass ratios, and thus a given plume brightness in Cassini images can correspond to a smaller mass in particles. Gao *et al.* found solutions in which the Enceladus photometry is consistent with either aggregates of small monomers or aggregates of larger monomers and solid particles. The inferred mass in particles is about 6 times smaller in the former case.

Since the start of Cassini's extended missions in 2008, hundreds of Cassini ISS images of Enceladus have been collected with the objective of deriving the detailed 3-D structure and particle content of the plume and how these properties vary over time. From such an analysis, it is then straightforward to determine how much mass is present at each altitude at any time, to compare these results to those of other Cassini instruments, and to estimate what mass in ice particles a future mission to Enceladus might collect during a typical fly-through of the plume at any altitude and at any time.

Our first goal is to present our work on a small collection of these images to derive quantities that are directly comparable to the results published by other Cassini Enceladus investigators and to account, if possible, for the apparent discrepancies, not by assuming particles of lesser mass as did Gao *et al.* (2016) but by accounting for the differing positions and times at which each observation was made.

Our second goal is to use our findings to examine how much organic material might be collected in a single traversal of the plume and whether or not it is reasonable to expect a detection of microorganisms. On Enceladus, ocean water rises under hydrostatic pressure through the ice shell; it is assisted in its final stretch to the surface by either boiling and/or the exsolution of volatiles as the liquid approaches the vacuum of space. Both processes involve bubbles: the former, water vapor bubbles; the latter, bubbles of volatile compounds such as CO_2_, CH_4_, and NH_3_. The plume ice particles are believed to be frozen droplets of ocean spray that were produced by the breaking of these bubbles (Postberg *et al.,* 2009, 2011). On Earth, where there are bubbles rising through natural waters, there will be bacteria and organic materials that adhere to the bubbles' surfaces and are consequently dragged up from below as the bubbles rise. They become enriched in the ejected spray that forms when the bubbles break. We refer to this phenomenon as bubble scrubbing; it is a complex process that has been known and studied for ∼90 years (Walls *et al.,* 2014). An examination of it makes it clear that a fly-through of Enceladus' plume may have more in common with a walk along a beach of breaking waves than might first meet the eye. We consider whether bubble scrubbing might be at work on Enceladus.

The layout of this paper is as follows. In Section 2, we describe our imaging data set and data reduction techniques, which are very similar, but not identical, to those described by Nimmo *et al.* (2014). In Section 3, we describe our photometric modeling efforts to derive the particle number and mass densities in the plume from ISS images. In Section 4, we review the previous remote and *in situ* observations of the plume and the estimates of various particle distribution properties derived from them. In Section 5 we compare all the previous estimates and those derived in this work, considering the times and geometries of each, in order to reconcile the seemingly discordant results. We also derive an estimate for the diurnally averaged mass production rate in solids. Finally, in Section 6, we estimate the bioload at putative seafloor hydrothermal vents on Enceladus, and from there consider the implications of our results for the future missions to Enceladus seeking evidence for extraterrestrial life. We conclude with Section 7.

## 2A. Challenges in Comparing Different Cassini Data Sets

It is a challenge to reconcile results obtained with very different measurement techniques (*i.e.,* remote sensing and *in situ*) used at different locations in the plume and at different times from different viewing geometries.

*In situ* observations, like those made by CDA and RPWS, allow a degree of spatial resolution and a local measure of conditions that are difficult, or even impossible, to obtain with remote sensing observations; the latter generally have much lower resolution and return a view that integrates all the material along the line of sight, resulting in measures of average properties. However, an accurate inference of the 3-D structure of the plume and its internal conditions from the limited *in situ* data that are collected during a few transects, and how those quantities might change over time, is very model-dependent.

Remote-sensing instruments like ISS and VIMS see sunlight scattered by small (∼1 μ) ice particles (Porco *et al.,* 2006); neither sees the vapor component. The plume is optically thin at visible and near-IR wavelengths; the measured integrated brightness is proportional to the instantaneous total surface area of all lofted ice grains along the line of sight that are efficient at scattering light at these wavelengths. Consequently, these observations produce an *instantaneous* particle density 2-D map of the entire structure as seen from one vantage point. With many images of the plume taken over a broad range of viewing angles and at various times, a 3-D picture of the distribution of the solid material in the plume and its variation with time can in principle be made in a way similar to medical tomography. One needs a model for light scattering, which for Enceladus' plume is Mie scattering, appropriate for the high phase angles involved.

The best possible situation occurs when both types of observations—remote and *in situ*—are made simultaneously, and each serves as a check and corroboration on the other. Unfortunately, on Cassini both observation types were never acquired at the same time. Therefore, to compare the results derived from different Cassini investigations, we must first account for the fact that the mass of the plume is spatially variable in three dimensions and on various timescales, and that differing observations were taken looking at a different aspect of the asymmetric plume. When the instrument techniques are so different, different sensitivities to different-sized particles may also play a role. No sound comparisons of observations, cross-instrument or otherwise, can be made without due consideration of these variabilities.

## 2B. ISS Data Selection and Reduction

Beginning with the start of Cassini's extended missions in 2008, several imaging campaigns were undertaken to support distinct scientific pursuits. One was a high-resolution survey of the SPT to determine the 3-D spatial distribution of geysers and their temporal variations, if any. The analysis of this set of ∼110 images revealed the presence of more than 100 discrete jets, with faint featureless material in between, erupting from four fractures crossing the SPT (Porco *et al.,* 2014). These geysers have an average spacing along the fractures of ∼5 ± 2 km (Helfenstein and Porco, 2015).

Another campaign called for images taken at moderate resolution over as large a time period as possible to (1) determine the 3-D structure of the plume and the sizes and concentrations of its icy particles and (2) monitor the time variability of the plume and determine its cause. The plume had been found in VIMS data to vary diurnally in brightness in a way that implicated tidal stresses (Hedman *et al.,* 2013). Initial results of this ISS campaign confirmed the diurnal variation and illustrated it was likely caused by the variation in extensional tidal stresses across the surface during the course of a day, with a phase lag whose origin was speculative (Nimmo *et al.,* 2014). The addition of more recent images has shown that the plume appears to be variable on longer timescales (Nimmo *et al.,* 2016; Ingersoll and Ewald, 2017), at least one of which is likely the result of a ∼4 year libration of the resonant argument of a corotation eccentricity resonance between Dione and Enceladus (Nimmo *et al.,* 2016); another ∼11 year libration may be present as well.

Determining the plume's structure and particle contents requires photometric modeling of the images to reduce plume brightness and its variation with solar phase angle to a suite of physical quantities: the size distribution, and volume number and mass densities per size bin of the particles, as well as their total abundance.

We leave for a later, more detailed and comprehensive paper our attempt to derive a phase function and, from that, a particle size distribution and search for variations with altitude and over time. In this work, for every chosen image, we perform our photometric analysis twice: once for a particle size distribution with an exponent of *q* = 3 differential (see Section 3) and another for *q* = 4. These bound the distributions that so far have been reported and/or studied by other plume investigators in the same portion of the plume studied here. The seven images used in this work for comparison with their results (Table 1) were selected to match the criteria already mentioned: first, they coincide close enough in time with observations made by other instruments so that the inherent long-timescale variability of the plume is removed as a possible cause for discrepancies; second, they were taken when Enceladus was near the same orbital position, or mean anomaly (MA), so that the large diurnal variation in mass was not a factor; and third, we selected from all the images meeting the first two criteria ones whose viewing angles, or subspacecraft longitudes on Enceladus, matched that of the observation being compared. This meant, for comparison with VIMS, we aimed to select an ISS image with the *same or nearly the same* Enceladus longitude; for comparison with CDA, we selected an ISS image having a subspacecraft longitude either 90° less or 90° greater than the ground track direction, so that the path of Cassini during CDA data collection—that is, the transect—sweeps across the image instead of in/out of it.

**Table 1.** ISS Images Used in This Work

*Image*	*Date*	*MA (°)*	*Phase (°)*	*Img scale (km/pixel)*	*SC_Lat (°)*	*SC_Long (°)*	*Sol_Lat (°)*	*Sol_Long (°)*	*Filter*
COMPARISON OF OBSERVATIONS									
N1516298600	2006-018T17:33:48	101	150.3	6.0	0.3	93.5	−18.9	297.1	CL1
N1634163141	2009-286T21:29:34	208	162.8	2.6	−0.3	298.8	1.0	101.6	CL1
N1639068152	2009-343T15:59:10	344	165.4	3.9	0.0	60.8	1.9	255.3	CL1
N1643192383	2010-026T09:35:51	269	161.0	5.4	−3.9	34.0	2.6	195.0	CL1
N1809922455	2015-130T03:12:24	100	153.7	2.2	0.1	87.2	24.6	257.8	CL1
N1816870210	2015-210T13:07:34	320	154.3	9.2	−0.3	314.9	25.0	142.2	CL1
N1829280199	2015-354T04:19:24	209	151.6	3.1	−1.1	269.3	25.7	74.5	CL1
EVALUATION OF ISS 178° IMAGE AND SECONDARY PEAK									
N1525413834	2006-124T05:33:24	63	156.9	13.5	0.3	95.8	−17.6	291.4	CL1
N1532053113	2006-201T01:47:21	66	148.0	11.1	0.4	109.7	−16.6	317.8	CL1
^*^N1537033403	2006-258T17:11:39	71	177.7	11.7	16.5	157.9	−15.9	340.1	CL1
N1830413543	2016-002T07:08:21	51	137.4	4.9	−0.8	64.5	25.7	280.4	CL1
SLAB MASS VS. MEAN ANOMALY									
N1516298600	2006-018T17:33:48	101	150.3	6.0	0.3	93.5	−18.9	297.1	CL1
N1516320030	2006-018T23:30:58	166	153.9	5.6	0.4	163.5	−18.9	2.3	CL1
N1516367311	2006-019T12:38:59	310	155.8	9.3	0.2	310.3	−18.9	146.0	CL1
N1525413834	2006-124T05:33:24	63	156.9	13.5	0.3	95.8	−17.6	291.4	CL1
*N1532048317*	*2006-201T00:27:25*	*52*	*147.9*	*11.5*	*0.4*	*95.1*	*-16.6*	*303.2*	*CL1*
*N1532050907*	*2006-201T01:10:35*	*60*	*147.9*	*11.3*	*0.4*	*103.0*	*-16.6*	*311.1*	*CL1*
*N1532053113*	*2006-201T01:47:21*	*66*	*148.0*	*11.1*	*0.4*	*109.7*	*-16.6*	*317.8*	*CL1*
N1537033403	2006-258T17:11:39	71	177.7	11.7	16.5	157.9	−15.9	340.1	CL1
N1608976498	2008-361T09:15:10	38	165.5	4.5	−8.4	29.8	−3.5	201.6	CL1
N1616348791	2009-080T17:05:50	107	164.6	3.9	−5.3	130.4	−2.2	297.0	CL1
N1634159832	2009-286T20:34:25	198	159.1	2.6	−0.3	292.4	1.0	91.5	CL1
N1634162091	2009-286T21:12:04	205	161.5	2.6	−0.3	296.8	1.0	98.4	CL1
N1634163141	2009-286T21:29:34	208	162.8	2.6	−0.3	298.8	1.0	101.6	CL1
N1637420790	2009-324T14:23:19	21	164.4	3.3	0.0	91.0	1.6	286.5	CL1
N1637420842	2009-324T14:24:11	22	164.3	3.2	0.0	91.1	1.6	286.7	CL1
N1639068152	2009-343T15:59:10	344	165.4	3.9	0.0	60.8	1.9	255.3	CL1
N1640477223	2009-359T23:23:31	303	160.7	3.3	−2.9	20.3	2.1	219.6	CL1
N1640478423	2009-359T23:43:31	307	157.6	3.2	−2.9	20.8	2.1	223.2	CL1
N1643187583	2010-026T08:15:51	254	156.1	5.7	−3.8	24.4	2.6	180.4	CL1
N1643192383	2010-026T09:35:51	269	161.0	5.4	−3.9	34.0	2.6	195.0	CL1
N1646167283	2010-060T19:57:10	304	155.2	6.3	0.2	84.9	3.1	240.3	CL1
N1646170883	2010-060T20:57:10	315	156.1	6.0	0.2	94.9	3.1	251.2	CL1
N1646174483	2010-060T21:57:10	326	156.7	5.6	0.2	105.3	3.1	262.2	CL1
N1646179299	2010-060T23:17:26	340	156.6	5.1	0.2	119.9	3.1	276.8	CL1
N1646182899	2010-061T00:17:26	351	156.0	4.8	0.3	131.5	3.1	287.7	CL1
N1646188916	2010-061T01:57:43	10	153.6	4.3	0.3	152.2	3.1	306.0	CL1
N1651010460	2010-116T21:16:13	252	161.9	6.0	0.0	43.6	3.9	206.0	CL1
N1652824635	2010-137T21:12:15	1	159.1	1.9	0.2	121.6	4.3	322.0	CL1
N1660401826	2010-225T13:57:51	333	162.2	1.9	−6.7	122.6	5.6	320.5	CL1
N1665895088	2010-289T03:51:34	95	156.7	4.0	−2.2	305.8	6.5	102.8	CL1
N1665899841	2010-289T05:10:47	109	161.7	4.0	−2.0	315.0	6.5	117.3	CL1
N1665902448	2010-289T05:54:14	117	164.8	4.0	−1.9	319.7	6.5	125.2	CL1
N1665903521	2010-289T06:12:07	120	166.1	4.0	−1.8	321.5	6.5	128.4	CL1
N1665903554	2010-289T06:12:40	121	166.2	4.0	−1.8	321.6	6.5	128.5	CL1

^*^ Very high phase, narrow-angle image used by IE11 (Ingersoll and Ewald, 2011).

• Image: Cassini image identification, with “N” standing for narrow-angle camera.

• Date: Time image was taken, in the format year–day of year–“T”–hours–minutes–seconds.

• MA: Mean anomaly of Enceladus at time the image was taken, where MA = 0° means that Enceladus is at its closest distance to Saturn and MA = 180° means that Enceladus is at its farthest distance from Saturn.

• Phase: Solar phase angle of observation, with 180° meaning exact forward scatter.

• Img scale: Width in km of 1 pixel, on the sky plane, for a given image.

• SC_Lat: Planetocentric latitude of Cassini spacecraft on Enceladus at time of observation, in degrees.

• SC_Long: Longitude of Cassini spacecraft on Enceladus at time of observation, in degrees.

• Sol_Lat: Planetocentric latitude of the Sun on Enceladus at time of observation, in degrees.

• Sol_Long: Longitude of the Sun on Enceladus at time of observation, in degrees.

• Filter: Name of the filter combination in which the image was taken. All images discussed in this paper were taken in the broadband clear filter.

In addition to trying to reconcile all the different data sets, we also measured the full range in variation of our derived quantities—such as how much mass can be collected during a single fly-through—over the course of an Enceladus day to assist those designing future missions back to Enceladus. For this purpose, we included images that were taken at the maximum and minimum of the diurnal variation, both early and late in the mission.

Finally, the only previous report of ice mass flux derived from ISS images was published by Ingersoll and Ewald (2011): 50 kg/s — 10 to 20 times larger than the fluxes derived from CDA's E7 and E21 flybys, respectively. To reconcile this particular conflict, we reduce ISS images of higher resolution but the same mean anomaly range as those used in IE11 and attempt to arrive at the source of the discrepancy.

**FIG. 1.**
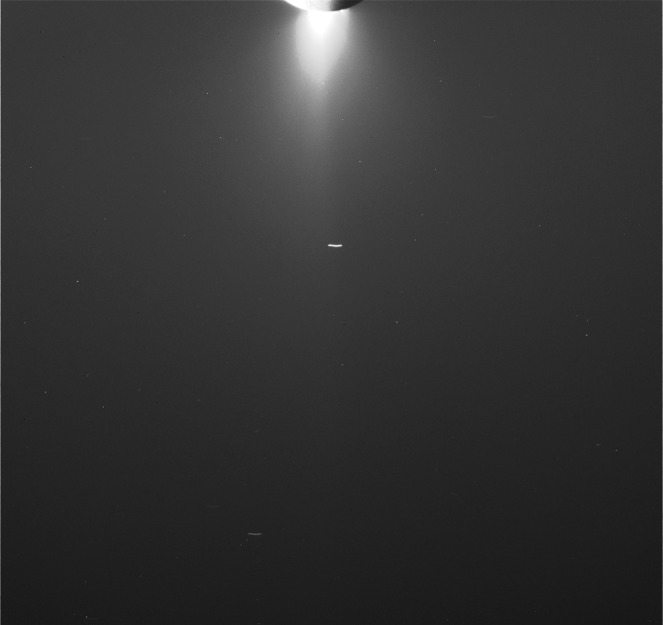
Calibrated version of Cassini NAC, clear-filter image N1634163141, taken on October 13, 2009, at 162.8° phase angle at an image scale of 2.6 km/pixel. Streaks are stars trailed during camera exposure.

All the images included in this work were calibrated with CISSCAL version 3.7; for example, see “Calibration Software” at http://pds-rings.seti.org/cassini/iss/software.html. Figure 1 is a calibrated version of one of the images used in this work, N1634163141. Once calibrated, all pixels have assigned to them an absolute measure of brightness, *I*/*F*.

The following steps were taken on every calibrated version of the images in Table 1 to reduce them to a proper form for photometric analysis.

### 2B.1. Pointing

To correct the pointing of the images, we followed the same procedure used in the work of Nimmo *et al.* (2014). The locations of limb points were calculated by finding the steepest gradients perpendicular to the limb, from moon to sky. These points were then fit to an ellipsoid generated from Enceladus' three principal axes. Both the sunlit crescent and unlit portion of the disk were used, when possible, in the fit. We excluded points that fell close to the plume, as well as points on the limb that were nearly the same brightness as the background E ring due to Saturn shine. (Depending on the viewing geometry, the part of the disk not illuminated by the Sun typically had a portion that was darker than the background E ring and a portion lit by Saturn shine that might or might not be brighter than the E ring.)

For this paper, we followed the same procedures as in the work of Nimmo *et al.* (2014) to subtract out the background in the images, with some exceptions. We increased the resolution criterion from an image scale of 1.8 km/pixel to 1 km/pixel, to increase the number of observations in our total collection over that used in our previous work. Though some of the much fainter extended plume does fall outside the higher-resolution image, in this study we concentrate on the core of the plume, which is well captured at 1 km/pixel. In the limit of pure ice, which we have assumed here, the absolute brightness of the scattered light at these high phase angles is partly due to diffraction and is therefore not significantly affected by composition, shape, or color of the particles, but only the ratio of particle size to wavelength. (Though the plume particles contain “contaminants” like salt and organic materials, the volume concentrations of these components are ∼1% [Postberg *et al.,* 2009], making pure ice a reasonable assumption.)

### 2B.2. Background fit and removal

As Enceladus is embedded in the E ring and the images are all taken at low subspacecraft latitudes on the moon, the E ring is seen more or less edge-on in our images. Therefore, to remove it, we create a synthetic image of the E ring as seen in the image and subtract it from the full image.

**FIG. 2.**
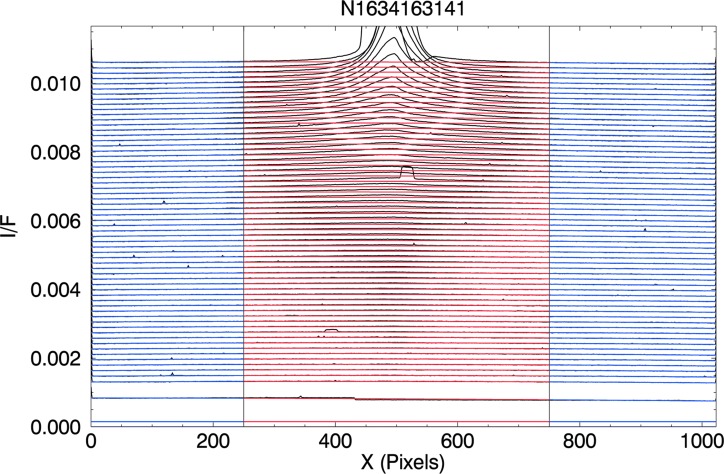
Scans of average *I*/*F* (black), taken across the image in Fig. 1, overlaid by the model scans (colored) derived from fitting the *I*/*F* scans to 1-D quadratics. The red portion of each scan was excluded from the fit. (See text for details.)

We begin by taking, for each image, a series of scans of *I*/*F* in the horizontal direction in Fig. 1, across the plume (our *x* axis), 64 in total each with a different Y position, and each 16 pixels in width. After median-filtering the scans to remove remaining cosmic rays, we fit the 64 individual background scans of *I*/*F* to 1-D quadratics. We exclude from these fits the portions of the scans that include any brightness from the plume itself and those small regions with surrounding background stars. In Fig. 2, the resulting background scans are colored curves overlaid on the *I*/*F* data from which they were derived. In the blue section of each curve, the background was determined by fitting to the underlying data. In the red section, the background was not fitted to the underlying data but instead was linearly interpolated between the blue sections on either side of the plume.^1^ To avoid edge effects, we neglect the nearest 5 pixels to the edges.

**FIG. 3.**
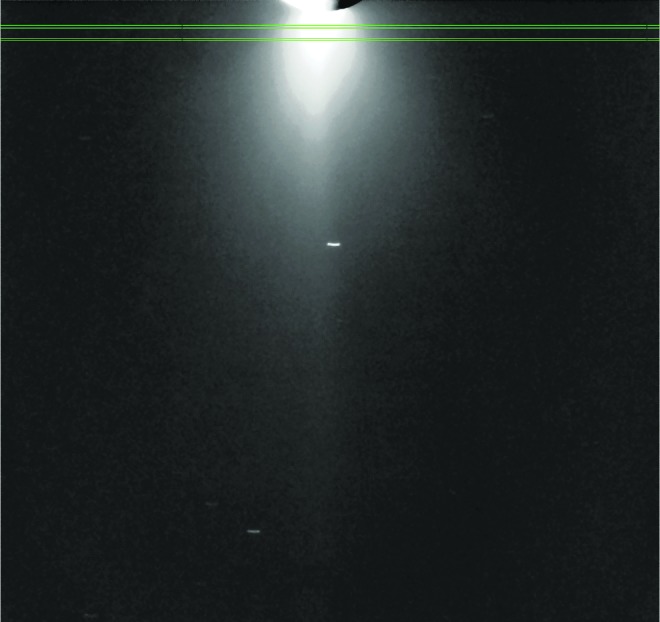
Two of our 20 slabs, each of 9 km vertical thickness, are shown laid on top of a reduced version of Fig. 1 in which the E ring has been subtracted and then median-filtered to remove or reduce the effect of cosmic rays and stars.

To finish the process, we fill in the horizontal regions between the scans in the vertical dimension, shown in Fig. 2, by linear interpolation in the Y direction, between scans. At this point, we have a synthetic, noise-free 2-D E ring to subtract out of the image, leaving a new image with zero contribution from the E ring. To this new image we apply a median filter, replacing the *I*/*F* value at each point with the median value of the *I*/*F* of the points in a 5 × 5 window centered on the point. This filter eliminates or significantly reduces stars and cosmic rays. Figure 3 is image N1634163141 background-subtracted in this way.

### 2B.3. Plume scans

Once the background has been subtracted, we integrate again in brightness across the entire image, cutting horizontally through and perpendicular to the plume's radial axis. The vertical thickness of the resulting slabs was set at 9 km. This means in our lowest-resolution image, with a scale of 13.5 km/pixel, the slab is a bit less than 1 pixel thick; in the highest-resolution images, it can be several pixels thick.

For each image, 20 such integrated scans were taken at altitudes above the south pole starting at 25 km and increasing by 25 km until the highest scan at 500 km. Figure 3 shows two such slabs, at 50 and 100 km.

We chose, as the vertical (lower and upper) boundaries for each integration, the *y*-axis positions that yield the proper absolute vertical thickness (in this work, 9 km) and the two *x*-axis (start and stop) locations in the scan direction as determined by one of two different criteria, whichever gave the widest plume width. First, we calculate the peak *I*/*F* of the plume and use 1/16 of this value as a threshold, finding the two *x*-axis positions (one on either side of the plume) along the scan where the plume emerges from, and then fades below, this value. Second, we use a constant value of *I*/*F* = 10^−5^ as the threshold and proceed as before, resulting in two more *x*-axis positions. The set of integration boundaries, X (start) and X (end), are then chosen from these two sets that give us the broadest plume. This procedure works well for both bright and faint scans, with the constant 10^−5^ threshold working well for bright scans and the 1/16 threshold working well for faint scans. These same horizontal (*x*-axis) boundaries serve as a consistent measure for the full extent of the plume's core, a quantity that is useful in our comparisons of plume dimensions among the various instruments. We also compute for each altitude a full-width half-maximum, FWHM, a critical quantity for computing volume number and mass densities and also determining plume shape.

**FIG. 4.**
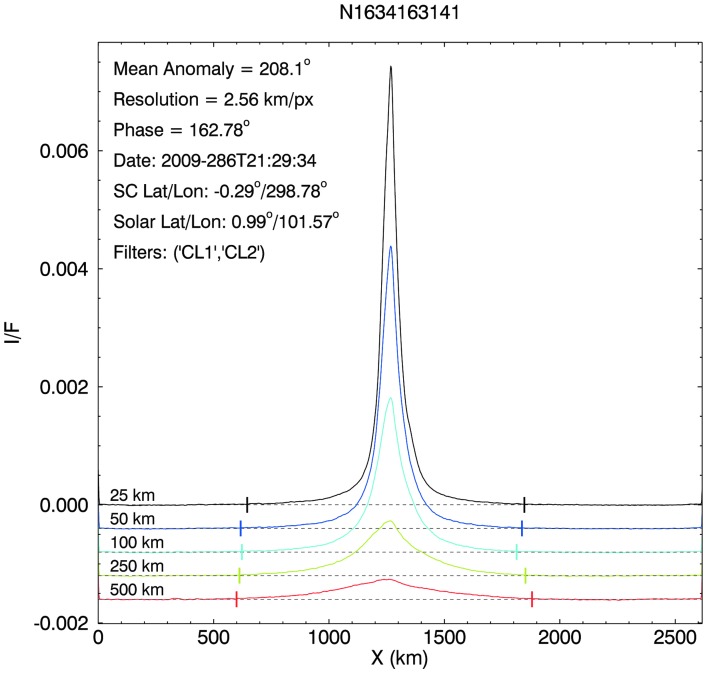
The scans of average *I*/*F* taken from the reduced and background-subtracted version (Fig. 3) of image N1634163141, with the zero points offset from each other for clarity. The *I*/*F* values in these scans have not been corrected for the falloff in NAC sensitivity over the course of orbital operations at Saturn, but corrected values have been used in all calculations in this paper. The vertical tick marks on the scans in this plot are the X boundaries used to delineate the full extent of the plume (see text).

Figure 4 shows five of our 20 scans across the plume in image N1634163141. Once the integration boundaries are chosen in both X and Y directions, the scan for any one altitude is created by beginning at the first X (start) and summing the *I*/*F* values at that X location in the Y direction, then averaging over the number of Y pixels in the sum to give, for that location, an average *I*/*F*. We then proceed along the scan to the next pixel in X, summing and averaging the brightness values in Y for that location and continuing in this fashion until the final X (end) boundary is reached. At this point, what is in hand is a string of values that are the average *I*/*F* at each X position at the center altitude of the scan that can be plotted to show the plume's horizontal profile (Fig. 4). When these averaged values are multiplied by the scan's absolute width in Y, they become ∫ (*I/F*) *dy*, where *dy* is the vertical distance in kilometers on the sky for each scan. These vertically integrated values, at each point in the scan, can be summed and multiplied by the X dimension of a pixel to yield the area under the curve ∫ (*I/F*) *dy dx*. This quantity is the area-integrated *I*/*F*, in units of *I*/*F* × Area (km^2^), and is directly comparable to the results presented by Nimmo *et al.* (2014).

### 2B.4. Narrow-angle camera (NAC) sensitivity correction

As the images were taken over a period of years at Saturn, following 7 years of travel to Saturn after Cassini's launch from Earth, changes in the camera sensitivity are to be expected. Based on calibration observations taken of the star Vega over the past 10 years, it has been determined that the NAC detector has degraded in sensitivity by 6% since 2004 (Ben Knowles, ISS internal document). To account for these changes, a correction factor was applied to the final integrated *I*/*F* values as follows, to derive corrected values, which we call *I*_corr_/*F:*

• Before August 6, 2004 (prior to decimal year *y* = 2004.596): No change; *I*_corr_/*F* = *I*/*F*
• August 6, 2004, to June 6, 2010 (2004.596 < *y* < 2010.429): *I*_corr_/*F* = *I*/*F* × [1.0 + 6.7183 × 10^−3^ (y−2004.596)]
• After June 6, 2010 (*y* > 2010.429): *I*_corr_/*F* = *I*/*F* × [1.0391871 + 4.0695 × 10^−3^ (y−2010.429)]

While our plotted scans in Fig. 4 have not been corrected in this way, our computations of plume contents (particle volume number densities, masses, etc.) have used *I*_corr_/*F* values.

## 3. ISS Photometric Modeling

The Enceladus plume is only easily visible at phase angles ≳130 degrees, indicating the number of small, roughly micron-sized diffracting particles vastly exceeds that of any much larger particles that might be present. Hence, we take the approach that a Mie scattering phase function is a very good approximation and fit, for a given altitude, the integrated *I*/*F* (described in the previous section) to the expected brightness from an assumed differential particle size distribution—either with exponent *q* = 3 or 4—for the appropriate phase angle.

To determine the mass of the particles in a slab within the plume, we must relate their measured reflectivity (*I*/*F*) to the light-scattering properties of the plume particles. If we treat a slab as an optically thin cloud of particles, the *I*/*F* is related to the optical depth *τ* by

\begin{align*}
 \frac { I }  { F } = \frac { { \tau { \omega _0 } \,\left\langle { P ( \alpha ) } \right\rangle } }  { 4 } \tag { 1 } 
\end{align*}

where *ω*_0_ is the single-scattering albedo and 〈*P*(*α*)〉 is the phase function of the plume particles at phase angle *α*. If we consider light passing through a uniform layer of particles (scatterers) of path length *L,* the optical depth of the slab can be expressed as *τ* = *nL*〈*C*_s_〉, where *n* is the number density of particles (in units of, *e.g.,* particles/m^3^) and 〈*C*_s_〉 is the average scattering cross section of a particle. The cross section *C*_s_ is often expressed as *C*_s_ = *Q*_s_*A*_p_, where *Q*_s_ is an efficiency factor and *A*_p_ is the cross-sectional area of a particle. For a spherical particle with radius *r*, *C*_s_ = *Q*_s_π*r*^2^. The quantity *nL* is the column density of particles, that is, the number of particles per unit area. The surface mass density Σ, that is, the mass per unit area, is simply *nL*〈*m*〉, where 〈*m*〉 is the average mass of a particle. Substituting in Eq. 1, we have

\begin{align*}
\sum = { \frac { 4 \left\langle m \right\rangle \frac { I }  { F }
} { { \omega _0 } \,\left\langle { P ( \alpha ) } \right\rangle
\,\left\langle { { C_ { \rm s } } } \right\rangle } }  \tag { 2 }
\end{align*}

The mass of the slab, *M,* is the surface density integrated over the area of the plume: *M* = ∫Σ*dA*. Finally, we set 〈*m*〉 = *ρ*_I_〈*V*_p_〉, where 〈*V*_p_〉 is the average volume of a particle and *ρ*_I_ is the internal density of a particle, to obtain

\begin{align*}
M = { \frac { { 4_ { \rho { \rm { I } } } } \,\left\langle { { V_ { \rm { p } } } } \right\rangle }  { { \omega _0 } \,\left\langle { { C_ { \rm { s } } } } \right\rangle \,\left\langle { P ( \alpha ) } \right\rangle } } \int { \frac { I }  { F } dA } \tag { 3 } 
\end{align*}

The average volume of a particle is given by

\begin{align*}
\left\langle { { V_ { \rm { p } } } } \right\rangle = { \frac { \int { { V_ { \rm { p } } } } ( r ) { \frac { dN }  { dr } } dr }  { { \frac { dN }  { dr } } dr } } \tag { 4 } 
\end{align*}

where *dN*/*dr* is the number of particles per unit volume with radii between *r* and *r* + *dr* and the volume of a particle of radius *r, V*_p_(*r*), equals 4π*r*^3^/3. Expressions analogous to Eq. 4 hold for 〈C_s_〉 and 〈*P*(*α*)〉.

### 3.1. Scattering cross section versus particle size

One issue of possible importance in the comparisons we attempt here among different types of data is that of detection sensitivity to particle size. Different instruments and measurement techniques may have different ranges of particle sizes that they can measure. For ISS, what is seen comes down to the scattering cross section of the particles.

**FIG. 5.**
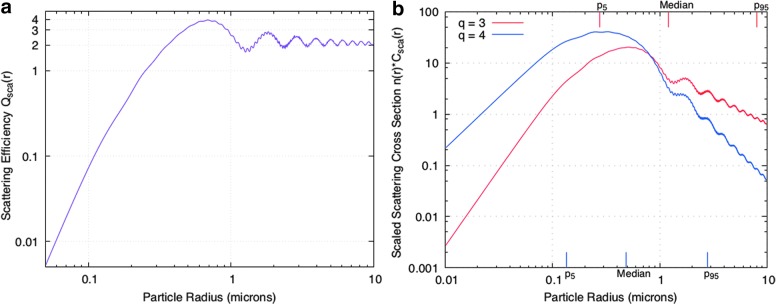
(**a**) The scattering efficiency *Q*_s_ of spherical particles is small for particles with radii, *r,* much less than the wavelength of light, λ, but increases as *r*^4^ in the “Rayleigh” regime. For water ice particles, *Q*_s_ increases up to a value of almost 4 for *r*∼λ and then oscillates, with the value approaching 2 for *r* >> λ. In this figure, we have taken λ = 0.651 μm, the effective wavelength for the NAC clear filter (Porco *et al.,* 2004). (**b**) Scaled scattering cross section of water ice particles for differential size distributions *dN*/*dr*∝*r^-q^*, with *q* = 3 (red) or 4 (blue). In each case *r*_min_ = 0.01 μm and *r*_max_ = 10 μm. Again, we have set λ = 0.651 μm. The values of the 5^th^, 50^th^, and 95^th^ percentiles for *q* = 3 are shown on the upper axis, while the corresponding values for *q* = 4 are shown on the lower axis. For *q* = 3, 90% of the scattering cross section resides in particles with radii between the 5^th^ percentile (0.274 μm) and the 95^th^ percentile (8.028 μm), *i.e.,* a factor of almost 30 range of sizes.

For particles with radius *r,* the scattering efficiency *Q*_s_ → 0 for *x* → 0, where *x* ≡ 2π*r*/λ and λ is the wavelength of light. Thus, the scattering cross sections of tiny particles are much smaller than their physical areas. (For pure ice particles at the wavelengths of the Cassini cameras, *Q*_s_ ∼ 0.1*x*^4^ for *x* << 1.) For particles much larger than the wavelength, *Q*_s_ → 2 for *x* →∞. In this limit, the scattering cross section *C*_s_(*r*) is proportional to the physical area of a particle. For particles larger than about the wavelength of light, *Q*_s_(*r*) oscillates as a function of *r* due to interference between diffracted and transmitted waves (van de Hulst, 1981; Chýlek and Zhan 1989), with the variations becoming smaller as the particle radius increases. This behavior is shown for water ice particles in Fig. 5a.

We have used Scott Prahl's Mie code (http://omlc.org/software/mie) to calculate the scattering cross section of water ice particles as a function of particle size. We choose distributions with *dN*/*dr* ∝ *r^−q^,* with *q* = 3 or 4, and λ = 0.651 μm, the central wavelength of the clear filter in the NAC; for size limits, we take *r*_min_ = 0.01 μm, *r*_max_ = 10 μm. Our results are shown in Fig. 5b. We find that for *q* = 3, the median particle size *r*_med_ (in terms of scattering cross section) is 1.206 μm, while 90% of the scattering cross section is in particles with radii between 0.274 and 8.028 μm. For *q* = 4, we find *r*_med_ = 0.488 μm, with 90% of the cross section in particles between 0.134 and 2.771 μm.

With all the size distribution parameters in hand, we use the freely available Mie code SPHER of Michael Mishchenko (https://www.giss.nasa.gov/staff/mmishchenko/ftpcode/spher.f), whose use was documented by Mishchenko *et al.* (2002, especially pp 160–164), to calculate the average (i) volume of a particle 〈*V*_p_〉; (ii) scattering cross section, 〈*C*_s_〉, averaged over the size distribution; and (iii) phase function 〈*P*(α)〉 for a variety of particle size distributions. We must determine these quantities because, in Eq. 3, the plume mass we infer is proportional to 〈*V*_p_〉 and inversely proportional to 〈*C*_s_〉 and 〈*P*(α)〉. We assume particles of pure water ice, with refractive index *n* = 1.3080 + 1.45 × 10^−8^ *i* at λ = 0.651 μm (Warren and Brandt, 2008). As our standard case, we assume *r*_1_ = 0.01 μm and *r*_2_ = 10 μm, with no particles smaller than *r*_1_ or bigger than *r*_2_. In this way, we are sure to cover the particle sizes with the greatest scattering cross sections for both size distributions. We take *ρ*_I_ = 0.9 g/cm^3^ and use the values returned by SPHER for 〈*P*(α)〉 at the phase angle for a particular image and for 〈*V*_p_〉 and 〈*C*_s_〉 for a given size distribution. The single-scattering albedos of our pure ice particles are 1 (to be precise, ≥ 0.999999).

For example, consider image N1639068152, taken on December 9, 2009, at a phase angle α = 165.4°. For *q* = 3, 〈*P*(α)〉 = 9.66, 〈*V*_p_〉 = 8.37 × 10^−3^ μm^3^, and 〈*C*_s_〉 = 5.81 × 10^−3^ μm^2^. For *q* = 4, 〈*P*(α)〉 = 10.73, 〈*V*_p_〉 = 8.68 × 10^−5^ μm^3^, and 〈*C*_s_〉 = 1.08 × 10^−4^μm^2^. Thus, for *q* = 3, ∫*I*/*FdA* = 1 km^2^ corresponds to 4 × 1 km^2^ × 0.9 g/cm^3^ × 8.37 × 10^−3^ μm^3^/(5.81 × 10^−3^ μm^2^ × 9.66) = 0.537 g km^2^ μm/cm^3^, or 5.37 × 10^6^g. For *q* = 4, ∫*I*/*FdA* = 1 km^2^ corresponds to 2.70 × 10^6^g, or about half the value for *q* = 3.

Select results derived from the photometric modeling of the ISS images we chose for this work are given in Table 2. There, we present two sets of transect values we inferred from ISS images for comparison with other data sets. (By “transect,” we mean a hypothetical fly-through of the plume equivalent to one of our *I*/*F* scans.) The first transect, which we here call *T*_1_, gives a typical value for the column density (number of particles per unit area) that a detector flying through the plume would sweep up. We calculate *T*_1_ = *n* × FWHM/10^7^, where *n* is the volume number density of particles, given in Tables 2 and 3, for three cases: particles with radii *r* > 0.5 μm, *r* > 1.5 μm, and for all particles (0.01–10 μm). The second transect, which we call *T*_2_, is, conceptually, a measure of the mass of particles per square meter that a detector would sweep up while crossing the plume. Following biological convention, we express *T*_2_ as *volume* per unit area in microliters per square meter (μL/m^2^), where 1 μL = 10^−3^ cm^3^ = 10^−9^ m^3^. We calculate *T*_2_ = *ρ* × FWHM/*ρ*_0_, where *M* is the mass of particles in the slab (again, in the volume *A* × *H*), and *ρ*_0_ = 1 g/cm^3^. We calculate *T*_2_ for all particles (to yield total collected mass) and for particles with *r* > 1.5 μm (to compare with CDA measurements). *T*_2_ is the volume per square meter in water ice (or liquid; we ignore the ∼10% difference in density between the two) that a collector would sweep out as it crosses the FWHM. We estimate that uncertainties, or real changes, in the shape of the plume result in uncertainties in our transect values of factors of O(2).

**Table 2.** Comparison of ISS, VIMS, and CDA Plume Observations

							*Volume (#/m*^3^*)*		*Transect (10*^7^*/m*^2^*)*				
*Instr*	*Obs*	*Date/Time*	*Alt (km)*	*S/C Long, or Ground-track +/− 90*°	*FWHM (km)*	*MA (°)*	(r *> 0.5 μ*)	(r *> 1.5 μ*)	(r *> 0.5 μ*)	(r *> 1.5 μ*)	*Transect (μL/m*^2^*)* (r *> 1.5 μ*)	*Transect (μL/m*^2^*) (all)*	*Optical depth τ*
VIMS	Cube 1	2005-331T15:06	50	109°	155^a^	105	879	190	13.6	2.9	—	—	
ISS ‘VIMS’	N1516298600	2006-018T17:33	50	94°	155	100	700–628	77–23	10.9–9.7	1.2–0.36	1.8–0.3	2.1–1.0	0.001
CDA	E7 Flyby	2009-306T07:42	100	∼70°	232	265	—	9	—	∼0.2^b,c^	—	—	
ISS ‘E7’	N1643192383	2010-026T09:36	100	34°	285	269	134–125	15–5	3.82–3.56	0.43–0.14	0.6–0.1	0.7–0.3	0.0003
CDA	E21 Flyby	2015-301T15:23	50	∼80°	46	103	—	65	—	∼0.3^b,c^	—	2	
ISS ‘E21’	N1809922455	2015-130T03:12	50	87°	100	100	867–782	94.4–29	8.67–7.82	0.94–0.29	1.4–0.2	1.6–0.8	0.0008
ISS—Early min	N1639068152	2009-343T15:59	50	255°	172	344	187–182	20–7	3.2–3.1	0.34–0.12	0.5–0.1	0.6–0.3	0.0003
ISS—Early max	N1634163141	2009-286T21:39	50	300°	92	208	3540–3340	385–124	32.6–30.7	3.54–1.14	5.2–0.8	6.2–3.0	0.003
ISS—Late min	N1816870210	2015-210T13:08	50	315°	258	320	107–96.8	12–3.6	2.76–2.50	0.31–0.09	0.4–0.1	0.5–0.2	0.0002
ISS—Late max	N1829280199	2015-354T04:19	50	269°	91	209	1990–1780	216–66	18.1–16.0	1.94–0.60	2.9–0.4	3.4–1.6	0.001

For ISS images, a range of values is given for, first, a *q* = 3 and, second, a *q* = 4 size distribution.

^a^ We set VIMS' FWHM equal to ISS' FWHM = 155 km.

^b^ CDA quotes values for *r* > 1.7 μ, not *r* > 1.5 μ, a distinction we ignore here.

^c^ Transect (column) densities are given by the product of peak volume density and, for VIMS, the FWHM of the brightness profile of the plume (which we set equal to the ISS FWHM), and for CDA, the estimated FWHM of the volume density profile measured during the traversal.

**Table 3.** Evaluation of Secondary Peak

							*Volume (#/m*^3^*)*		*Transect (10*^7^*/m*^2^*)*			
*Instr*	*Obs*	*Date/Time*	*Alt (km)*	*Long (°)*	*FWHM (km)*	*MA (°)*	(r *> 0.5 μ*)	(r *> 1.5 μ*)	(r *> 0.5 μ*)	(r *> 1.5 μ*)	*Transect (μL/m*^2^*)* (r *> 1.5 μ*)	*Transect (μL/m*^2^*) (all)*
I/E^*^	N1537033403	2006-258T17:11	50	158	211	71	535–1590	58–59	11–33	1.2–1.2	1.8–0.9	2.1–3.3
ISS ‘I/E’	N1525413834	2006-124T05:33	50	96	176	63	518–474	56–18	9.1–8.3	1.0–0.3	1.8–0.9	1.7–0.8
ISS ‘I/E’	N1532053113	2006-201T01:47	50	110	222	66	440–387	48–14	9.7–8.6	1.1–0.3	1.6–0.2	1.8–0.8
ISS ‘I/E’	N1830413543	2016-002T07:08	50	65	133	51	1120–933	122–34	15–12	1.6–0.5	2.4–0.3	2.8–1.2

^*^ Subspacecraft latitude = 16.5^o^; pixel scale = 11.7 km.

We also, in Table 2, include a measure of peak optical depth in the plume, *τ*, at the appropriate altitude, derived from our photometric modeling.

## 4. Previous Remote and *In Situ* Plume Observations

### 4.1. VIMS

At the moment, the only remote sensing data that have been reduced to yield particle number densities with altitude in the plume are those collected by the Cassini VIMS experiment in its near-IR channel (Hedman *et al.,* 2009). This channel measures spectra at 256 wavelengths between 0.85 and 5.1 μm with a typical spectral resolution of 0.016 μm. For the data discussed here, the *spatial* resolution was 0.25 × 0.5 mrad, forming in all a map of the spectral properties of a given scene known as a “cube.” These VIMS data on Enceladus' plume were collected on November 27, 2005, at a solar phase angle of 161°, a forward-scattering geometry that brightens tiny particles. They were reduced to three cubes, each the average of several cubes (to increase signal to noise), and were obtained at mean ranges of 131,700 km (Cube 1), 139,500 km (Cube 2), and 158,300 km (Cube 3) from Enceladus over the course of ∼4.5 h.

From the spectral information in the data and assumptions about light scattering characteristics of the particles at high phase, Hedman *et al.* (2009) calculated particle number densities at a range of altitudes in the plume from ∼50 to ∼280 km for 1, 2, and 3 micron–sized particles, the sizes present in any measurable numbers that VIMS could detect. (Perhaps, due to low resolution or low sensitivity, VIMS was not sensitive to particles greater in size than 3 microns, though the *in situ* instruments detected them.) These values, plotted as the total number of particles in a horizontal slab slicing the plume at a given altitude, per size bin (in meters) and per meter vertical slab thickness are shown in Fig. 5 in the work of Hedman *et al.* (2009). We utilized their values for a 50 km altitude in Cube 1.

To convert these into volume number densities or transect (column) densities requires knowledge of the basic size and shape of the plume. The plume's horizontal dimension was not resolved in these VIMS data. Hence, we assign to the VIMS observation the same FWHM measured in the ISS comparison image (N1516298600), 155 km, since it was taken at approximately the same mean anomaly and subspacecraft longitude and only 1.5 months later. We assume for the present study that the plume's horizontal cross section is roughly spherical, though there is evidence that it is somewhat asymmetrical.

We recast the data plotted in Fig. 5 of Hedman *et al.* (2009) using the same approach that we used in deriving the volume and column (transect) number densities from ISS images: that is, we take the integrated slab number density, that is, total number of particles integrated across particle sizes, per meter vertical thickness, and divide by the surface area of the slab, taken to be *A* = π × (FWHM/2)^2^. These numbers are given in Table 2.

### 4.2. CDA E7

Dust detections by both RPWS and CDA during the E7 (November 2, 2009) 91 km altitude flight directly over the south pole and diametrically through the heart of the plume were compared by Ye *et al.* (2014). Both instruments consistently found a particle size distribution to be well described by a simple differential power law with *q* ∼ 4 at this high altitude, within a particle size range of 1.7–10 μ, and unlike VIMS, with no break in the particle size distribution. Both found peak particle volume densities in the range of *n* = 6–10/m^3^.

For the most recent analysis of the CDA High Rate Detector E7 results, we referred to a poster presented at the Fall 2016 AGU meeting in San Francisco (Kempf *et al.,* 2016). This instrument has two modes: one measures the mass of each collected particle, and the other measures size. For the closest flybys during which CDA acquired its best particle data, the latter mode was used. Their reported peak volume number density was *n* = 9/m^3^ for *r* > 1.7 μ and *n* ≈ 0.2/m^3^ for *r* > 6.5 μ. The reported size distribution was a simple power law, with no peaks, with *q* ≈ 3.5; the total modeled mass production rate was 2.7 kg/s.

Since the CDA volume number densities are measured in a traversal of the plume, they can easily be converted to the total number of particles per square meter, in a given size range, collected during one traversal of the plume by finding the area under the density profile. An estimate of its FWHM, taken from the figure in their poster, was ∼30 s; the flyby speed was 7.74 km/s, yielding a FWHM ∼ 232 km. Consequently, the area under the curve *H* = *n* × FWHM = 2 × 10^6^/m^2^ (Table 2).

### 4.3. CDA E21

We treat the CDA E21 (October 28, 2015) 50 km altitude flyby results in the same way as the E7 flyby; our source for these data is also the work of Kempf *et al.* (2016). This flyby was faster (8.5 km/s), off-center, and did not diametrically cut the plume through its center. And it was lower than E7, at an altitude of 50 km. Not surprisingly, the peak density was higher—*n* ≈ 65/m^3^—for a total collected volume of ∼2 μL/m^2^. Our estimate of its FWHM was 5.4 s, which yields 5.4 s × 8.5 km/s = 46 km. Thus, *H* = *n* × FWHM = 3 × 10^6^/m^2^ (Table 2). The reported modeled mass flux was 5 kg/s.

### 4.4. ISS IE11

None of the non-ISS data were taken when the plume was at its maximum strength, at MA ∼208°. However, we know now that the particular mean anomaly range covered by the 2006 images used by IE11 to derive a 50 kg/s mass flux captured Enceladus as it was traversing a part of its orbit in which the plume exhibits a secondary peak that reaches a brightness almost as large as it does at its maximum.

**FIG. 6.**
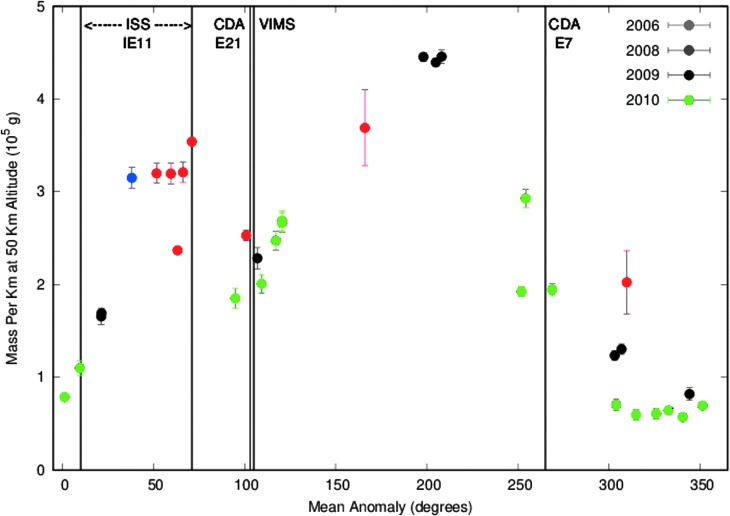
The mass in slabs of 1 km vertical thickness at an altitude of 50 km as a function of Enceladus' mean anomaly, obtained from our photometric analysis of the NAC clear-filter images taken early in the mission listed in Table 1, for our *q* = 3 distribution. (See text for the details of the mass calculation.) Indicated are the mean anomalies of Enceladus at the times of the VIMS, CDA E7, and CDA E21 results. We also indicate the range of MA spanned by the images used by IE11 to derive the 50 kg/s mass flux. The error bars reflect photon noise, *i.e.,* $\sqrt N$ noise, where *N* is the number of photons detected by the camera. The systematic uncertainty in the absolute calibration of *I*/*F,* which we estimated to be 7% in Nimmo *et al.* (2014), has not been included here.

Figure 6 is a plot of the 50 km altitude slab masses we derived, from a series of images taken *early* in the mission (Table 1), as a function of the mean anomaly of Enceladus at the time the image was taken^2^. This secondary peak is obvious and present every time that ISS captured the mean anomaly range from ∼10° to 90°; it peaks at MA ∼45–65°. The first hint of this peak's existence in ISS data was a single high datum at MA ∼38° presented in the work of Nimmo *et al.* (2014); that it was likely part of a broader “hump” became clearer when examined against the VIMS observations of the plume (Hedman *et al.,* 2013). Its existence was not known at the time of the IE11 work. This feature was discussed by Helfenstein and Porco (2015), who attempted to explain it by cross-cutting fractures that might also open and close with a period of a day, but no conclusion could be reached. As of this writing, its cause is unknown.

We chose several ISS images taken at approximately the same MA as the very high phase angle (178°) NAC used by IE11 (henceforth called 178NAC): two were taken within 134 days of the 178NAC. An almost continuous sequence of images, designed by the authors of this paper especially to cover this peak, was taken 10 years later, in 2016. We use one of the 2016 images in our analyses here. (The full 2016 set covering the rise of the secondary peak is discussed in a follow-on paper.) We also reduce and evaluate the 178NAC using our methods.

In Table 1, we list the observational parameters of all these images; in Table 3, we present the results of our analyses of them. Our transect values for capture on a 1 m^2^ collector at 50 km altitude for all the 2006 images, including 178NAC—either for all particle sizes or *r* > 1.5 μ, regardless of *q*—all place this peak at ∼1/3 the values for the main peak as the latter was observed early in the mission (N1634163141). Our volume densities for the same size ranges differ by a factor of ∼1/7 from the early maximum, also regardless of *q*. This peak is indeed a secondary one.

We note, however, in Table 3, that for the 178NAC, the *q* = 4 volume densities and transect values in the *r* > 0.5 μ size range increase from their lower phase angle values, while the corresponding *q* = 3 values remain nearly the same. Unlike the other three images taken at lower phase angle, the extraordinarily high phase of the 178NAC falls within that regime of the plume's phase function where a more top-heavy size distribution (like *q* = 3) can become photometrically distinguishable from a bottom-heavy one (like *q* = 4). This suggests that we could use the approximate consistency in the volume densities for the *q* = 3 distribution across the low and high phase angle images in Table 3 as an indication that a *q* = 4 distribution can be ruled out. We take up this issue in the next section.

In Fig. 6 we indicate the mean anomalies of all the previous plume investigations mentioned above.

## 5. Comparisons among All Plume Investigations

As mentioned in Section 2A, we need to account for known temporal variabilities and differences in plume dimensions and measurement locations to derive accurate conclusions from the comparison of different data sets. We know from our earlier work that the integrated brightness of the plume can be 3 or 4 times greater at maximum than minimum over the course of an Enceladus day (Nimmo *et al.,* 2014) and at its maximum can change by a factor of 2 over long timescales (Nimmo *et al.,* 2016). Also, stochastic variations caused, as one example, by geysers turning on and off during the time frame of the observations may still be present and causing any remaining unpredictable variations in our measured widths and modeled particle densities and plume masses.

Consequently, we deliberately chose for comparison with each earlier observation at least one ISS image that was close in mean anomaly, in time, and in subspacecraft longitude. In comparing ISS to the CDA measurements—a special case—images were chosen that were close *to perpendicular* to the groundtrack so that the images captured the same aspect of the plume sampled by the *in situ* trajectory. The pairings are given in Table 2, along with the relevant quantities derived from published results.

ISS and VIMS, though covering different, but overlapping, wavelength regimes, are both remote sensing instruments and subject to the same inherent limitations and uncertainties in taking photometric measurements and deriving particle distributions from them. Considering the stochastic variations in brightness (and hence derived mass), even at the same mean anomaly, that seem inherent in the plume's behavior (*e.g.,* Nimmo *et al.,* 2014), and obvious changes—both systematic and stochastic—in the plume's width, the ISS- and VIMS-derived densities shown in Table 2 are encouragingly close—within a factor of 2.5 at *q* = 3. If we interpret them at face value, our results for particles with *r* > 1.5 μ agree more closely with VIMS for a *q* = 3 distribution than for *q* = 4. This is marginally consistent with the conclusions reached by VIMS, that between 1.5 μ < *r* < 3.5 μ, the distribution has *q*≈3. Our results for 0.5 μ < *r* < 1.5 μ are not so selective and could agree with either distribution. The VIMS and ISS comparison observations were acquired at MA ∼100°, when the plume's strength is ≤0.5 of its maximum but not in its lowest state (see Fig. 6).

The E7 flyby occurred when the plume was near MA = 265°, a state comparable to that at MA = 100° but at twice the altitude and later in the mission than VIMS; both conditions result in a decrease in plume mass. And as expected, comparing volume and transect column densities for *r* > 1.5 μ, we see that the CDA E7 values are lower than those of VIMS by large factors: 10 to 20. The same comparison between CDA E21 and VIMS—both taken at the same altitude, MA, and close in subspacecraft longitude—yields better agreement, at least for volume densities: for *r* > 1.5 μ, VIMS finds 190/m^3^ and CDA E21 65/m^3^, different by a factor of 3. We consider a difference of a factor of 3 in volume densities to be reasonably good agreement, all things considered. (The transect densities clearly differ by a factor of 10 because on top of the factor of 3 in the volume densities, there is a factor of 3 in the FWHM.)

ISS and CDA results show even better agreement. Comparing volume and transect column densities for *r* > 1.5 μ, CDA E7 values fall approximately midway between the ISS *q* = 3 and *q* = 4 values. Encouragingly, Kempf *et al.* (2016) reported *q* = 3.5 at an altitude of 100 km. For E21, the lower flyby, the CDA *r* > 1.5 μ transect density that we have computed falls very close to the ISS *q* = 4 values, while the CDA transect *total volume* density given explicitly by the CDA team, and presumably more carefully calculated than our values, falls closer to *q* = 3 ISS results. No conclusion is given by Kempf *et al.* (2016) about *q* for this 50 km flyby, though at 50 km at the same mean anomaly, VIMS sees a distribution that is closer to *q* ≈ 3.

The suggestion from these comparisons thus far is that at 50 km altitude, *q* ≈ 3, and it may increase to 3.5 or 4 at 100 km. This would be consistent with the expected gravitational stratification in particle size (Hedman *et al.,* 2009). We continue this investigation into the value of *q* in the following paragraphs.

We compared with special interest our analysis of images falling on the secondary peak, including the 178NAC, with that of IE11. The sequence of wide-angle camera (WAC) images used by IE11 to determine the particle size distribution and the total mass of particles in the plume, and hence produce the 50 kg/s result, spans mean anomalies from ∼10° to 68°. The 178NAC was the highest phase of all, followed the WAC sequence, and falls at MA = 71°. It is now clear that all these images caught the plume as it was inherently increasing in brightness. At the same time, the phase angle varied from 175° to 178°, extremely high phase during which brightening by diffraction from the particles in the plume is expected to be large and very discriminating of particle sizes and their distribution. Hence, the phase angle dependence and total brightness are sensitive indicators of the total mass of solid material in the plume. Without accounting for the inherent brightening and increasing mass of the plume, the quantities derived from a photometric analysis of this image set—that is, size distributions, phase functions, plume mass, and hence mass production rate—will be in error.

**FIG. 7.**
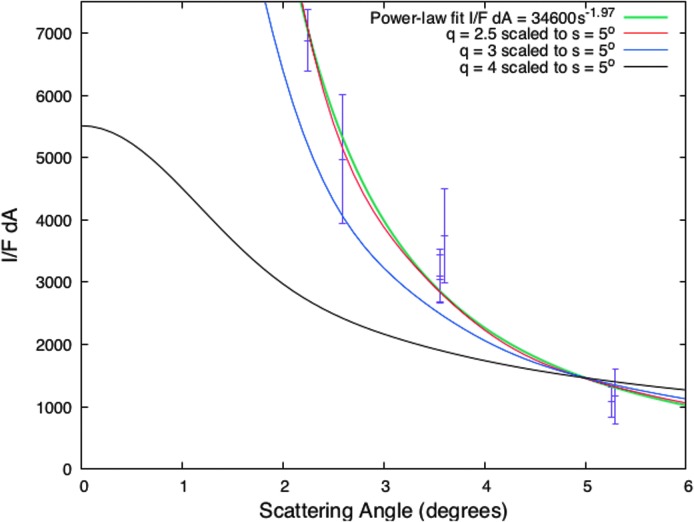
A graph showing the integrated *I*/*F* in a slab for *q* = 3 and *q* = 4 size distributions, and our power-law fit to the discrete values of the phase function derived in IE11 from the 2006 very-high-phase WAC observations. We find a fit of *q* ∼ 2.5 (green). A *q* = 2.5 Mie phase function is shown in red. (See text for details.)

To illustrate, we compare in Fig. 7 the discrete phase function IE11 derived from their data with the *q* = 3 and *q* = 4 functions we assumed in this work. Their points can be represented by a power law in scattering angle (SA) which, nominally, corresponds to *q* ∼ 2.5. (The smaller the value of *q,* the more *I*/*F* increases as the scattering angle decreases.) Within the uncertainties, their curve is indistinguishable from *q* = 3 and seems to rule out *q* = 4. However, the highest phase angle (lowest scattering angle) point suggests that *q* ∼ 2.5 may be correct. This implies more large particles and therefore more mass than what we infer for our *q* = 3 distribution, and certainly more mass than the CDA E7 and E21 flybys which find *q* = 4.

As the phase angle *α* increases from 174.7° to 177.75°, corresponding to scattering angle SA = (180° − *α*) decreasing from 5.30° to 2.25° in Fig. 7, the integrated *I*/*F* increases by a factor *f* ∼ 6–7. If plume activity remained constant with time, this rapid change with scattering angle would indicate a top-heavy size distribution for the plume particles—that is, a small value of *q* (such as the value of 2.5 we derived from Fig. 7)—and hence comparatively more large particles and more mass. This was the conclusion of IE11; they derived the largest reasonably precise mass flux in solids of all the investigations. However, while scattering angle decreased in the images examined by IE11, the mean anomaly of Enceladus increased from 10° to 71°. Over this range of mean anomalies, which includes the secondary peak, images taken at smaller phase angles, and plotted in Fig. 6, indicate that the plume brightens intrinsically by a factor *f*_I_ ∼ 3. If the brightening seen by IE11 is due to both the decrease in scattering angle and the increase in mean anomaly, the change due to the phase function of the plume particles could be as small as *f*/*f*_I_ ∼ 2. In this case, *q* could be as large as 4.

However, a more detailed look at the trend of *I*/*F* with mean anomaly in Fig. 6 leads us to conclude that, more likely, *q* ∼ 3. The secondary peak has a maximum at MA ∼45–65° (Section 4). In other words, though Fig. 6 indicates that the plume brightness rises rapidly as MA increases from 10° to 38° (the latter is the blue point), the brightness is nearly constant for 45° ≤ MA ≤65°. The flatness near the peak can be seen in Fig. 6 as the points at MA = 52°, 60°, and 66° with nearly identical values of mass per kilometer. All three points derive from images taken the same day in 2006, less than 2 months before the images analyzed by IE11, at a phase angle of 148° (see the italicized lines in Table 1). If we assume that the plume brightness is also constant with mean anomaly for 56° ≤ MA ≤ 71°, we can use IE11's highest phase angle points to infer *q.*^3^

Specifically, the ratio of the integrated *I*/*F* value reported by IE11 at *α* = 177.6° (SA = 2.4°) to the integrated *I*/*F* at *α* = 176.4° (SA = 3.6°) is 2.1 ± 0.1. For Mie phase functions with *q* = (2.5, 3, 3.5, 4), this ratio is (2.5, 2.1, 1.7, 1.4). The agreement between the measured ratio and the model ratio for *q* = 3 indicates that the index of the size distribution must be near 3. This supports our finding in Section 4 that the volume densities at different phase angles are more consistent with each other for *q* = 3 than for *q* = 4.

Further work is needed to disentangle these effects, but it is very likely that the 50 kg/s mass flux quoted by IE11 is too large.

Finally, in comparing the results from various investigators, it is important to note the region of the plume being sampled. The very much brighter core of the plume has a FWHM brightness that varies with altitude. At 50 km altitude, which is the region sampled by this work, it varies from ∼90 to 200 km. Beyond that there are extensive wings, the most distant parts of which do not become apparent until very high phase angles are reached, for example, the ∼175° ISS WAC images. The region we chose here to study is the same region that was sampled by the CDA during E7 and E21, and measured by VIMS in the work of Hedman *et al.* (2009).

We save a detailed derivation of the mass production rate for our follow-on paper, but we estimate it here by determining the mass in a 1 km thick slab at 50 km averaged over an orbit and calculating how much mass may be passing through it. From Fig. 6, we find the diurnally averaged mass (*i.e.,* “area under the curve”), to be ∼2.5 × 10^5^ g/km. This calculation assumes *dN*/*dr* ∝ *r^−q^*, with *q* = 3 between *r*_1_ = 0.01 μm and *r*_2_ = 10 μm, and *ρ*_I_ = 0.9 g/cm^3^. If we instead assume *q* = 4, while keeping *r*_1_, *r*_2_, and *ρ*_I_ unchanged, the mass we infer is about half that shown in Fig. 6 for all the images except 178NAC. For 178NAC, the mass we infer for *q* = 4 is approximately 1.5 times greater than that for *q* = 3.

The mass in a slab consists of particles recently ejected from Enceladus rising and also falling through the layer. If we consider only the gravitational attraction of Enceladus on a particle and assume the particles are launched vertically, the velocity, *v,* at altitude *y* is related to the velocity at the surface, *v*_0_, by

\begin{align*}
 { v^2 } = v_0^2 - 2GM \left( { \frac { 1 }  { { { R_ { \rm { E } } } } } - \frac { 1 }  { { { R_ { \rm { E } } } + y } } } \right) \tag { 5 } 
\end{align*}

where *GM* = 7.2027 ± 0.0125 km^3^ s^−2^ is the gravitational mass of Enceladus and *R*_E_ = 252.1 ± 0.1 km is the radius of Enceladus. The classical escape velocity from Enceladus is *v*_e_ = (2*GM*/*R*_E_)^½^ = 239.0 m/s. To reach altitude *y,* a particle must have *v*^2^ > 0, which implies *v*_0_ > *v*_0*m*_, where

\begin{align*}
 { v_ { 0m } } = \sqrt { { \frac { 2GMy }  { { R_ { \rm { E } } } ( { R_ { \rm { E } } } + y ) } } } \tag { 6 } 
\end{align*}

For *y* = 50 km, *v*_0*m*_ = 97 m/s. Particles are ejected from Enceladus with a range of velocities. To motivate our discussion, we identify three velocity regimes.

(1) Particles launched with *v*_0_ < *v*_0*m*_ do not contribute to the mass of the layer.
(2) Particles launched with *v*_0*m*_ < *v*_0_ < *v*_e_ contribute to the mass of the layer twice—once going up, once coming down.
(3) Particles launched with *v*_0_ > *v*_e_ contribute to the mass of the layer once—going up.^4^

The mass in the slab, *M,* due to each subset of particles is

Case 1: Zero
Case 2: *M* = *dm*/*dt* × 2 *dt,* where *dt,* the time for a plume particle to cross the slab, equals the slab thickness divided by the velocity of the particle.
Case 3: *M* = *dm*/*dt* × *dt*

To evaluate *dm*/*dt,* we must estimate which fraction of the particles are ejected in each of the three velocity ranges. We consider the distribution of launch velocities, *f*(*v*_0_), which is defined so that *f*(*v*_0_)*dv*_0_ is the fraction of particles ejected with velocities between *v*_0_ and *dv*_0_. IE11 investigated three functional forms—Gaussian, exponential, and Lorentzian—for *f*(*v*_0_), and favored the exponential distribution, *f*(*v*_0_) = 1/*v*_★_ exp(−*v*_0_/*v*_★_), with *v*_★_ = 90 m/s.^5^ This fit was carried out for the plume out to altitudes of 14 *R*_E_. Ingersoll and Ewald (2017) fit the same three functions to plume data at altitudes between 50 and 200 km and found a larger value of *v*_★_, 210 m/s, for the characteristic velocity of the exponential distribution.^6^ For an exponential distribution, the fraction of particles with velocities between *v*_1_ and *v*_2_ is exp(−*v*_1_/*v*_★_) − exp(−*v*_2_/*v*_★_). Then the fraction of particles *f*_1_, *f*_2_, and *f*_3_ in the velocity regimes above are

Case 1: *f*_1_ = 1 − exp(−*v*_0*m*_/*v*_★_)
Case 2: *f*_2_ = exp(−*v*_0*m*_/v_★_) − exp(−*v*_e_/v_★_)
Case 3: *f*_3_ = exp(−*v*_e_/*v*_★_)

For *v*_★_ = 90 m/s, (*f*_1_, *f*_2_, *f*_3_) = (0.66, 0.27, 0.07), while for *v*_★_ = 210 m/s, (*f*_1_, *f*_2_, *f*_3_) = (0.37, 0.31, 0.32).

The time for a plume particle to cross a slab of thickness 1 km is *dt* = 1 km/*v,* where *v* is related to *v*_0_ by Eq. 6. Using these considerations, we can relate *M* to *dm*/*dt* as follows:

\begin{align*}
M = { \frac { dm }  { dt } } \times 1 \ { \rm { km } } \left( { 2 \int_ { { v_ { 0m } } } ^ { { v_ { \rm { e } } } } { d { v_0 } \frac { { f ( { v_0 } ) } }  { v } + \int_ { { v_ { \rm { e } } } } ^ \infty { d { v_0 } \frac { { f ( { v_0 } ) } }  { v } } } } \right) \tag { 7 } 
\end{align*}

or, substituting *f*(*v*_0_) = 1/*v*_★_ exp(−*v*_0_/*v*_★_),

\begin{align*}
M = { \frac { dm }  { dt } } \times { \frac { 1 \, { \rm { km } } }  { { v_ { \star } } } } \left( { 2 \int_ { { v_ { 0m } } } ^ { { v_ { \rm { e } } } } { d { v_0 } \frac { { \exp ( - { \frac { { v_0 } }  { { v_* } } } ) } }  { v } + \int_ { { v_ { \rm { e } } } } ^ \infty { d { v_0 } \frac { { \exp ( - { \frac { { v_0 } }  { { v_ { \star } } } } ) } }  { v } } } } \right) \tag { 8 } 
\end{align*}

yielding

\begin{align*}
M = { \frac { dm }  { dt } } \times { \frac { 1 \, { \rm { km } } }  { { v_ { \star } } } } \left( { 2 \int_ { { v_ { 0m } } } ^ { { v_ { \rm { e } } } } { d { v_0 } { \frac { \exp ( - { \frac { { v_0 } }  { { v_ { \star } } } } ) }  { \sqrt { v_0^2 - v_ { 0m } ^2 } } } + \int_ { { v_ { \rm { e } } } } ^ \infty { d { v_0 } { \frac { \exp ( - { \frac { { v_0 } }  { { v_ { \star } } } } ) }  { \sqrt { v_0^2 - v_ { 0m } ^2 } } } } } } \right) \tag { 9 } 
\end{align*}

and finally

\begin{align*}
M = { \frac { dm }  { dt } } \times { t_ { { \rm { eff } } } } \tag { 10 } 
\end{align*}

where

\begin{align*}
 { t_ { { \rm { eff } } } } = { \frac { 1 \ { \rm { km } } }  { { v_ { \star } } } } \left( { 2 \int_ { { v_ { 0m } } } ^ { { v_ { \rm { e } } } } { d { v_0 } { \frac { \exp ( - { \frac { { v_0 } }  { { v_ { \star } } } } ) }  { \sqrt { v_0^2 - v_ { 0m } ^2 } } } + \int_ { { v_ { \rm { e } } } } ^ \infty { d { v_0 } { \frac { \exp ( - { \frac { { v_0 } }  { { v_ { \star } } } } ) }  { \sqrt { v_0^2 - v_ { 0m } ^2 } } } } } } \right) , \tag { 11 } 
\end{align*}

In Eq. 11, *t*_eff_ is an effective residence time in a kilometer-thick slab for *all* particles launched, including those moving too slowly to ever reach the slab. For instance, for *v*_★_ = 90 m/s, 26.9% of the particles are launched at speeds between *v*_0*m*_ and *v*_e_. These particles traverse the slab twice, once going up, once coming down. During each traverse, the average time spent in the layer is 14.6 s. A total of 7.0% of the particles are launched at speeds greater than *v*_e_; these particles spend an average of 3.4 s in the layer. (The remaining 66.1% of the particles never reach an altitude of 50 km.) The effective residence time is then *t*_eff_ = 0.269 × 2 × 14.62 s + 0.070 × 3.41 s = 8.11 s. For *v*_★_ = 210 m/s, *t*_eff_ = 0.309 × 2 × 12.42 s + 0.320 × 2.72 s = 8.54 s. In fact, for all values of *v*_★_ between 90 and 210 m/s, *t*_eff_ is between 8.11 and 8.96 s. For *M* = 2.5 × 10^5^ g, that is, *M* = 250 kg (for *q* = 3), the corresponding values of *dm*/*dt* are 30.8 kg/s and 27.9 kg/s; for *q* = 4, *dm*/*dt* is 14.6 and 13.2 kg/s, assuming no change in the particle size distribution with altitude. In keeping with our earlier inference that at 50 km *q*≈3, we take the diurnally averaged mass production rate, for the 2006–2010 timeframe, to be 29 ± 7 kg/s, smaller by a factor of ∼2 than that favored by IE11. (The uncertainty arises from the photometric errors in our analysis and our estimate of the inherent stochasticity in the plume's brightness.) Hedman *et al.* (2009) found in analysis of VIMS observations that the most massive particles are relatively more abundant at lower altitudes than above, that is, the *q* of the distribution becomes smaller below. Accounting, then, for those heavier particles that are present at lower altitudes, do not reach 50 km altitude, but are not accounted for by our altitude-independent size distribution will increase our derived rate. Thus, 29 kg/s is at present a lower limit that pertains to the early part of the Cassini mission, and specifically for *q* = 3 at all altitudes up to 50 km.

Our choice of 10 μ for the upper size cutoff has a potentially significant and growing effect on our results as *q* becomes smaller than 4, because the larger particles carry the greater mass. For example, for *q* = 3, there is equal mass in equal size intervals; ignoring particles between 10 and 20 μ would be ignoring approximately half the mass, assuming these particles are actually present. For *q* = 4, the effect is small; the mass only increases by ∼9% if we increase the upper cutoff from 10 to 20 μ. We chose a 10 μ upper limit because the *in situ* instruments report none that are bigger than this limit, though theirs is a statistical limit set by the size of the detector.

There could be, of course, a true sharp, upper size limit for particles in the vents, arising naturally from the physics of bubble creation and bursting and/or the number density and size distributions of the resulting droplets above the water table in the fractures, and other environmental conditions, all of which are now poorly known.

The total derived mass depends also on the lower size limit. For particles derived from droplets created by bursting bubbles, this number could naturally be set by a minimum size in the bubble-forming process owing to the energy cost of producing very small bubbles that do not immediately collapse (Gonnermann and Manga, 2007). There are also particles in Enceladus' plume, as small as nanometers in size, that are believed to be due to homogeneous nucleation from vapor. In choosing a lower limit of 0.01 microns, we are assuming these components are distributed with the same *q,* that is, no break in the size distribution, which may not be correct. For *q* = 3, the mass derived is insensitive to the lower cutoff *r*_1_ for *r*_1_ ≤ 0.1 μm, while for *q* = 4, the mass increases as we make *r*_1_ smaller because copious tiny particles contribute mass but do not scatter light effectively. In Tables 4 and 5, we show the sensitivity of our derived mass loss rates to different assumptions about the values of *r*_1_ and *r*_2_ for *q* = 3 and 4.

**Table 4.** Sensitivity of Mass Loss Rate to Particle Size Distribution, Phase Angle 157°

	r_*1*_	r_*2*_	dM/dt *(minimum)*	dM/dt *(maximum)*
q	*μm*	*μm*	*kg/s*	*kg/s*
3	0.01	5	15.8	17.5
**3**	**0.01**	**10**	**27.9**	**30.8**
3	0.01	20	49.9	55.1
3	0.001	10	27.9	30.8
3	0.1	10	27.7	30.5
3	0.5	10	37.1	40.9
3	1	10	57.4	63.3
4	0.01	5	12.0	13.3
**4**	**0.01**	**10**	**13.2**	**14.6**
4	0.01	20	14.4	16.0
4	0.001	10	17.6	19.4
4	0.1	10	8.9	9.8
4	0.5	10	13.4	14.8
4	1	10	34.1	37.6

**Table 5.** Sensitivity of Mass Loss Rate to Particle Size Distribution, Phase Angle 178°

	r_*1*_	r_*2*_	dM/dt *(minimum)*	dM/dt *(maximum)*
q	*μm*	*μm*	*kg/s*	*kg/s*
3	0.01	5	21.2	23.4
**3**	**0.01**	**10**	**27.9**	**30.8**
3	0.01	20	51.4	56.7
3	0.001	10	27.9	30.8
3	0.1	10	35.4	39.1
3	0.5	10	26.9	29.7
3	1	10	27.4	30.2
4	0.01	5	50.5	55.7
**4**	**0.01**	**10**	**48.5**	**53.6**
4	0.01	20	52.6	58.1
4	0.001	10	64.7	71.4
4	0.1	10	32.4	35.7
4	0.5	10	23.3	25.7
4	1	10	24.1	26.7

## 6. Sample Collection and Expectations

We can now ask the question: How much material would we expect a future spacecraft to collect if it flew through the plume at a close-approach altitude of 50 km? Obviously it depends at what time during an Enceladus day the fly-through occurs. To bound the problem, we also examined ISS images that were taken at the maximum (MA≈208°) and minimum (MA ∼ 330°) extremes of the brightness (and mass) variation, both early in the mission when the plume is more massive and later when it is more feeble. These results from this work are also given in Table 2.

We find from our modeling that at 50 km altitude, the column density can vary by a factor up to 10 between the plume's minimum and its maximum states, regardless of *q*. For our preferred *q* ∼ 3, the total variation between minimum plume state late in the mission and maximum plume state early in the mission is ∼0.5 to 6 μL/m^2^ (Table 2), for an average of ∼3 μL/m^2^. A collecting plate 20 cm on a side would capture (assuming 100% efficiency) a range of ∼0.02–0.24 μL in particles of all sizes; the average plume state yields a transect sample size of ∼0.1 μL. How much material—microbes or organic matter—might this yield?

### 6.1. Microbes

With the discovery of evidence of hydrothermal activity within Enceladus' ocean, it is plausible that microbes could thrive in oceanic vents on the Enceladus seafloor, where such activity would be concentrated, in the same way we find thriving ecosystems at Earth's seafloor vents. In particular, the detection of H_2_ in the Enceladus plume by Cassini's mass spectrometer (Waite *et al.,* 2017) has been interpreted to indicate a source of food for such organisms, in particular methanogens, who live off the reaction 4H_2_ + CO_2_ → CH_4_ + 2H_2_O. Based on this H_2_ detection and a hydrothermal circulation model powered by Enceladus' observed energy flux, and assuming (i) only 10% of the geothermal power is transported by hydrothermal flow, (ii) methanogenesis is the sole metabolic pathway, (iii) H_2_ concentration in the Enceladus vents is equal to that measured in the Lost City vents on Earth, and (iv) complete conversion of H_2_ into biomass, microbial concentrations have been estimated to be as high as 10^9^ cells/mL at the vent fluids and about 10% of that in the Enceladus plume (Steel *et al.,* 2017, in this issue).

Here, we take a different, less optimistic approach. We begin with a calculation similar to that originally proposed by Jakosky and Shock (1998)—the ratio of the geothermal fluxes through the seafloors of both bodies—and then estimate biomass on Enceladus by scaling the microbial concentrations found at terrestrial ocean vents by the ratio of the bodies' geothermal energy fluxes. The geothermal flux through the seafloor from the core is given by *E*/*A; E* is the geothermal power coming out of the core, and *A* is the surface area of the seafloor. If we assume that the fraction of this flux that is available on Enceladus to produce geochemical energy, and from there biomass, is identical to the fraction at Earth's ocean vents that creates biomass, then we can simply take the ratio of their energy fluxes to be the ratio of their bioloads; that is,

\begin{align*}
{C_{{ \rm{EN}}}} / {C_{{ \rm{EA}}}} \,\sim \,\left( {{E_{{ \rm{EN}}}} / {A_{{ \rm{EN}}}}} \right) / \left( {{E_{{ \rm{EA}}}} / {A_{{ \rm{EA}}}}} \right)
\end{align*}

where *C* is the concentration of microbes.

The greatest source of energy on Enceladus is tidal energy dissipated in the interior. We know that at the very least there is ∼5 GW of energy observed radiating from the tiger stripe fractures (Spencer *et al.,* 2013). Calculations of how much total tidal energy could be dissipated in the core have yielded values as high as 20 GW (Tobie *et al.,* 2017). Based on the recession of the orbit of Enceladus, the tidal dissipation within the interior is expected to be ∼10 GW (Lainey *et al.,* 2017). We take 10 GW as representative value for the total geothermal power leaving Enceladus' core. Since tidal energy is largely dissipated at the poles, even within the core, we take the seafloor on Enceladus to be that under the SPT sea, bounded by 60° latitude. The relevant surface area will be *A*_EN_ = *A*_SPT_ ≈ 2π*R*_C_^2^ [1 − cos(30°)] ≈ 0.3 × 10^11^m^2^, where *R*_c_≈200 km is the radius of Enceladus' core. Thus, on average and ignoring factors of O(2), the geothermal flux through the SPT seafloor will be *E*_EN_/*A*_EN_≈0.1 W/m^2^.

The geothermal flux on Earth is very spatially variable and is greater through the ocean floors than the continents. The average Atlantic ocean floor heat flux, again ignoring factors of O(2), is ∼0.1 W/m^2^ (Sclater *et al.,* 1980). Thus, we find *C*_EN_/*C*_EA_≈1.

Assuming the fraction of geothermal flux emerging at seafloor hydrothermal vents versus the more widely distributed diffusive heat flow into the ocean is similar between Earth and Enceladus, and that the conversion of hydrothermal energy into biomass is equivalent on both worlds, then the concentration of organisms supported by geothermal energy turned to biomass will be comparable for the two worlds.

The microbial ecosystem at the ultramafic, alkaline, hydrothermal vent, Lost City, is quite diverse metabolically, and microbial cycling of sulfur and methane are the dominant active biogeochemical processes (Brazleton *et al.,* 2006). Oxidizing organisms are found at Lost City in the cooler, lower-pH, exterior portions of the chimneys where O_2_ dissolved in the cool seawater circulates along with hydrothermal fluid. But only anaerobic chemoautotrophs are found in the hotter, higher-pH, more vigorously venting interior parts of the chimneys where the greatest abundances of H_2_ and CH_4_ are also found.

We do not expect photosynthesis, or the products of photosynthesis such as O_2_, to be a dominant microbial energy source on the dark seafloor of Enceladus. However, the presence of oxidants is not implausible. Slow convection of the ice shell could deliver surface oxidants, produced by radiolysis, to the ocean. Another, not insignificant source of oxidants may be produced by the radiolysis of water circulating through the rocks in the upper part of the moon's core (Bouquet *et al.,* 2017). These two sources of oxidants, as well as radiolytically produced H_2_, could provide another familiar source of chemical energy for microbes, that is, sulfur-oxidizing and sulfur-reducing reactions, which are common at Lost City (Brazelton *et al.,* 2006). No sulfur-bearing compounds have been confidently detected in the plume of Enceladus, though Cassini instruments were not optimized to see them and some sulfur-containing products would quickly become chemically bound in the ocean and never make it to the plume. It is perhaps too premature to rule out the presence of oxidizing or sulfur-metabolizing microbes on Enceladus. Thus, we assume here that the hydrothermal vents on Enceladus and on Earth are geologically, chemically, and even to some degree metabolically similar.

The cell concentrations in samples taken from various sites within the Lost City vent field, both in the high-pH, H_2_- and CH_4_-rich, hot inner chimney and subsurface locales, as well as the cooler, lower-pH, exterior regions where hydrothermal fluids are diluted with seawater, are ∼10^5^/mL (Brazleton *et al.,* 2006). Similar concentrations have since been found at other ultramafic, low-temperature seafloor vents, such as Quest (Perner *et al.,* 2010) and Von Damm (Reveillaud *et al.,* 2016). Consequently, our 1:1 scaling, assuming energy and metabolic partitioning is the same on both worlds, tells us that Enceladus' oceanic hydrothermal vents should have *C*_EN_ ∼ 10^5^/mL.

The Enceladus ocean is estimated to be ≥30 km thick (Thomas *et al.,* 2016), though it is possible that it is as deep as 50 km (Čadek *et al.,* 2016). Any organisms born at the ocean bottom would have to rise that distance before beginning the ascent through the ice shell. Biogeochemicals originating at Earth's seafloor vents and carried through the ocean in warm, hydrothermal plumes have been detected at distances >4000 km from their sources (*e.g.,* Resing *et al.,* 2015). Such plume features are now known throughout all Earth's ocean basins (German *et al.,* 2016). The observation of Si-bearing compounds in the Enceladus plume particles, taken as evidence of the moon's hydrothermal activity (Hsu *et al.,* 2015), is in itself a demonstration that Enceladus' seafloor material may traverse the deep ocean and rise through the ∼25 km ice shell, to be expelled eventually in the plume at high enough concentrations to be detected by Cassini. As long as the moon's rotation period is sufficiently shorter than the rise time through the ocean, and there are no strong turbulent currents in the ocean, thermal plumes originating at the seafloor on a rotating planet can remain more or less collimated through Coriolis forces, which are strongest in the polar regions. Enceladus' rotation period is 33 h. The rise time through the ocean of warm, hydrothermal materials has been calculated using a 2-D GCM, applied to the evolution of a single plume, to be between 11 and 55 days (Steel *et al.,* 2017, in this issue). Thus, it is possible that the thermal plumes will remain more or less intact up to the base of the ice shell.

If hydrothermal activity is widespread on the seafloor, leading to a high spatial density of thermal plumes, and the vorticity in the plume is strong enough to prevent entrainment of seawater (Maxworthy and Narimousa, 1994), and the lifetime of organic materials (microbes included) against decay is sufficiently long, then the continued delivery of these materials from the seafloor below may build up a layer at the ice/water interface where concentrations of the delivered materials are in fact enhanced over their source values. It is an important problem in the study of the transport of biologically significant materials on Enceladus and worthy of future study.

For the time being, we take the dilution of 10:1 as estimated by Steel *et al.* (2017, in this issue); this gives *C*_EN_≈10^4^/mL at the base of the ice shell. If this concentration is unchanged in the conduits that lead to the surface, a 0.04 m^2^ collecting plate with 100% efficiency would collect ∼1 organism in a single transect at 50 km for the “average” state of the plume and a sample size of 0.1 μL. This is insufficient to detect with a microimager.

### 6.2. Bubble scrubbing

However, there are good reasons why inherent concentrations of microbes and compounds of biological or environmental interest *in the plume* could be many times higher than their bulk ocean concentrations. Geysering is a process in which bubbles, formed by volatiles coming out of solution, are a dominant feature. Bubbles rising through a column of water can scrub the column of organic matter and microbes (viruses, bacteria, cells, toxins, etc.) because these substances become physically attached to the film at the air/vapor interface and disperse into a spray of tiny droplets when the bubble reaches the surface and breaks. This is a phenomenon that is ongoing in all natural bodies of water such as oceans, rivers, lakes, and ponds; has been known and studied for over 90 years; and on which there is an extensive literature. A high bacterial count on the surfaces of natural waters and in the spray produced by breaking waves is common on Earth, and rough sea conditions can increase concentrations of particular types of compounds in aerosols above the sea by several orders of magnitude compared to those found in the bulk ocean (O'Dowd and de Leeuw, 2007).

Studies done in laboratory experiments have reported enhancement factors (EFs), above the bulk water microbial concentrations, that vary by orders of magnitude, from single digits to thousands (*e.g.,* Carlucci and Williams, 1965; Blanchard and Syzdek, 1970), with at least one reported measurement of EF ∼ 30,000 (Blanchard and Syzdek 1972). The range is large because there are many variables that come into play in the process. Composition, surface properties, morphology (size, shape), and even the health of an organism have been found to be correlated with observed EF. Temperature, inorganic content (*e.g.,* salinity), and other environmental variables are also EF correlates. In reverse, the properties of the adsorbed matter can alter the behavior of the bubbles and the aerosols it produces. For instance, bubbles with surface contamination can linger longer at the surface before breaking than can uncontaminated bubbles (Wallis *et al.,* 2014).

Nonetheless, some basic physical principles underlying bubble scrubbing (also referred to as scavenging) and the interactions between a bubble and its environment—inorganic, organic, and biological—are now understood. We describe the basics here. (For a review, see Wallis *et al.* [2014].)

#### 6.2.1. Life of a bubble

**FIG. 8.**
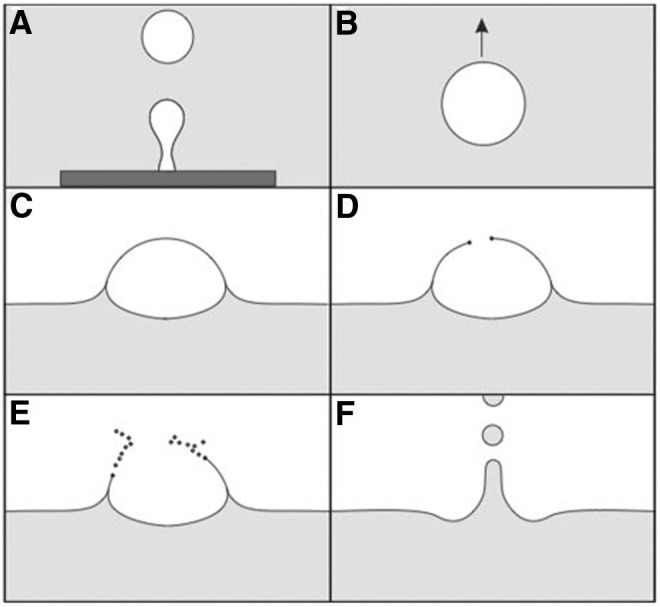
The various stages in the life of a bubble. (**A**) Formation; (**B**) ascent; (**C**) at free surface, followed by drainage; (**D**) hole nucleation and film retraction; (**E**) production and launch of film drops; (**F**) production and launch of jet drops. Figure from Wallis, Bird and Bourouiba (2014) *Integrative and Comparative Biology,* volume 54, number 6, pp. 1014–1025, doi:10.1093/icb/icu100. Reproduced with permission from Oxford University Press.

The general life of a bubble is depicted in Fig. 8. A bubble begins when a volatile comes out of solution, or air is forced into a fluid, and begins its ascent through the fluid column driven by buoyancy. As the top of it meets the air/water interface at the surface, a thin film forms the upper, roughly hemisphere-shaped boundary between entrapped vapor in the bubble and the ambient vapor above. As the bubble continues to rise, the film becomes thinner due to drainage of fluid downward along the sides of the film, until finally the surface tension holding the film together is overcome, and the bubble bursts. In the bursting process, the film begins to retract and produce droplets that are expelled upward. These are called film droplets. The final stage of a bubble's life is the approach to equilibrium of the air/water interface at the bottom of the (now broken) bubble, which results in the development of a central rise of fluid, much like the central peak created (and solidified in place) by a crater-forming impact on a planetary body. This fluid peak, or jet, turns into droplets; these are called jet droplets. Both types of droplets compose the aerosols that accompany any process that punctures the top of a natural body and entrains air, such as rainfall, waterfalls, and fountains. They also compose the spray that is produced by the breaking of waves on the shore of Earth's oceans.

If the origin of most of Enceladus' plume particles are liquid droplets originally sourced in its subsurface ocean, then it is of great relevance that film and jet droplets can in principle be produced by the same bubble yet have two distinct size regimes and spectra, maximum contaminant loads, and relative abundances, all depending on the size of the original bubble (see Table 6).

**Table 6.** Size Distributions of Film and Jet Drops in Laboratory Experiments

*Drops*	*Bubble size range*	*Drop #'s*	*Drop size range*
Film	*D* < 0.5 mm	0	0.1 μ < *d* < … 30 μ and maybe higher;size distribution can vary by 2 orders of magnitude^a^
	0.5 mm < *D* < 3 mm	100–200	
	3 mm < *D* < 6 mm	200–1000	
Jet	*D* < 0.3 mm	5 or more	*d* < 30 μ^b^
	0.2 mm < *D* < 1.8 mm	up to 5	20 μ < *d* < 180 μ^b^
	*D* > 6 mm	only 1	*d* > 600 μ^b^

^a^ Droplets with *d* < ∼(5–10 μ) are mostly film drops (Blanchard and Syzdek, 1982).

^b^ ∼5–20% bubble size; jet drops seldom vary in size by more than a factor of several.

**FIG. 9.**
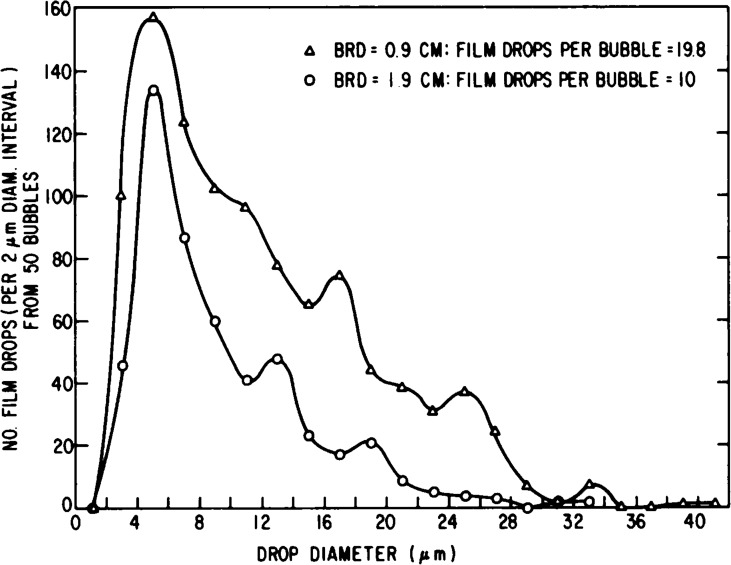
Size distribution found for film droplets produced from fifty 1.7 mm diameter bubbles, in two experimental setups with two different bubble rise durations (BRD). Size distribution remains essentially the same. Figure from Blanchard and Syzdek (1982) *Applied and Environmental Microbiology,* May 1982, p. 1001–1005 Vol. 43, No. 5, 0099-2240/82/051001-05$02.00/0. Reproduced with permission from American Society for Microbiology.

The number of film drops increases with increasing bubble size. While in lab experiments there are no film drops produced by bubbles with diameters *d* < 0.5 mm, the number produced by bubbles between 0.5 and 3 mm in diameter can range from 100 to 200 (Mason, 1954) and up to 1000 for a 6 mm bubble. The drops' ultimate sizes depend on the size of the bubble, the bubble's surface tension (including the effects of its surfactants), and the thickness of the film at the time of bursting (which also depends on its contaminants). In lab experiments, the sizes of film drops have been found to vary over 2 orders of magnitude, from <0.1 μ to beyond 30 μ. Figure 3 of Blanchard and Syzdek (1982) reproduced as our Fig. 9 shows a size distribution obtained from 50 bursting bubbles of ∼1.7 mm diameter in an experiment to investigate film drops. It is sharply peaked at *d* ≈ 5 μ; more than half the drops have *d* < 10 μ.

Contrary to film drops, the number of jet drops decreases with increasing bubble size (Blanchard and Syzdek, 1982). Jet drops tend to be 5–20% the size of the original bubble, and their size distribution is much narrower, varying by approximately factors of 3–5. Bubbles with *d* < 0.3 mm produce five or more jet drops, those with 0.2 mm < *d* < 1.8 mm produce up to five jet drops, but those with *d* > 6 mm produce only one.

It is not uncommon for a bubble to form daughter bubbles of a smaller size and thus create a cascade of jet drops, even if the original bubble was >6 mm in diameter.

In summary, for a given bubble size, film drops are smaller and more numerous than jet drops. Larger bubbles (*d* > 3 mm) tend to be dominated by film drops, and smaller bubbles tend to be dominated by jet drops. The top jet drop is always the biggest and has the greatest concentration. In the past it was believed that, in natural waters, bubbles in the size range of 1–3 mm are most important for film drop production, and that aerosol drops in the size range from 5 to 10 μ produced in natural waters are predominantly film drops (Blanchard and Syzdek, 1982). However, in marine waters, bubbles rarely exceed 1 mm in diameter at the surface, suggesting that jet drops are the main constituents of the cloud of aerosols above the liquid surface as shown by more recent high-speed micrography measurements (Liger-Belair *et al.,* 2001; Schmitt-Kopplin *et al.,* 2012). The issue of drop size distributions is still unclear.

#### 6.2.2. Physics of attachment

If the bulk water is not pure but contains bits of either inorganic or organic matter (including microorganisms), these can become attached to the vapor/water interface of the bubble as it rises. Whether or not this occurs depends primarily on the solubility of the material and its surface activity, that is, how it affects the surface tension of the bubble. Surfactants, materials with high surface activity, reduce the bubble's local surface tension and attach efficiently (provided the saturation limit has not been reached, in which case the bubble surface behaves rigidly). Consequently, bacteria that release surfactants or act like them readily attach to bubbles. Hydrophobic molecules also attach preferentially over hydrophilic ones. Cell hydrophobicity is often one of the most important physio-chemical factors in the attachment of organisms to bubbles (Dahlbäck *et al.,* 1981). The attachment preference of pigmented bacteria is reducible to the hydrophobic nature of their cell surfaces (Hermansson *et al.,* 1982) in some cases and their tendency to produce surfactants in others (Blanchard and Syzdek, 1978).

The relationship between the abundance of organic matter on the bubble's surface and that of microbes is a complicated one, but if organic matter is already present, bacteria will readily attach to it. The concentration of cations and their influence on the expression of cell hydrophobicity (Fattom and Shilo, 1984) may modify the adsorption of cells to interfaces and their EFs in droplets. The addition of salts increases the resultant drop EF. Also, water-insoluble compounds have a higher attachment efficiency than water-soluble ones, so the ratio of water-insoluble to soluble compounds will differ from the bulk ocean. In general, high concentrations of microbes are associated with high concentrations of particulate matter. Furthermore, the concentration of bacteria and organic matter on the bubble's surface once it reaches the surface, assuming it is not already saturated with attached matter, will increase even more because of the film of scum (matter and microbes) already there from previous bursting bubbles that did not fully eject their cargo. However, on Enceladus this last step may not occur if a film of organic matter is prevented from forming by any process which significantly disturbs the top of the water column, like rapid gas exsolution or turbulent mixing of water in the fractures (Kite and Rubin, 2016).

Aside from the primary properties mentioned above, other secondary characteristics that affect how efficiently matter and microbes will attach to rising bubbles are as follows: how much material is already present, how long it takes the material to attach (related to its surface chemistry) compared to the time it takes it to slide off the interface and back into the bulk fluid, the length of time the bubbles rise through the column, whether or not the saturation limit has been reached, and the bulk concentration of the water. The longer a bubble ascends, the more matter and microbes it will adsorb.

In any one environment, like the sources of the geysers on Enceladus, in which we might safely assume the environmental conditions—temperature, pressure, and chemistry—are more or less uniform for all particles, we could expect the biggest drops, which are likely to be jet drops from the largest bubbles, to have the greatest concentration of bacteria, but film drops to be more numerous if bubble sizes are big enough to create them.

Several factors may work in our favor and increase the organic and microbial loads on the bubbles emerging from the top of the water column on Enceladus. The greatest EFs have been found for the smallest bulk concentrations (Blanchard and Syzdek, 1972). In laboratory experiments, an initial bulk microbial load of 7 × 10^3^/mL resulted in the top jet drop of 0.7 mm diameter having a maximum EF of ∼30,000 in 5 s of ascent. It could be that all bubbles have reached their full EFs on Enceladus and these EFs are high. Second, salts and even organics are present in Enceladus particles (Postberg *et al.,* 2009, 2017), which enhances attachment and increases EF. Third, while ongoing bubble-scrubbing could eventually deplete the water of organic matter, it is plausible that the diurnal tidal cycle hydraulically pumps water in and out of the fractions, forcing water into the ocean during compressional periods and allowing ocean water to refill them during extensional periods. This could keep the bulk concentration of microbes and organics in the fractures at a steady level, especially considering that the volume of water in the fractures is negligible compared to that in the global ocean. Finally, the exsolution of insoluble plume compounds, like CH_4_, possibly occurs very deep, perhaps even at the seafloor. Thus, bubble scrubbing could take place over the entire vertical ascent, through the deep ocean and then through the conduits in the ice shell leading to the surface. Expected microorganism sample sizes for a single 50 km transect increase from 1 up to 10^3^ organisms. This is within range of current microimaging capabilities (Bedrossian *et al.,* 2017, in this issue).

#### 6.2.3. Organic matter

As we already mentioned, molecules with high surface activity (*e.g.,* surfactants) and especially hydrophobic molecules will readily attach to a rising bubble and therefore be chemoselectively concentrated in the resulting aerosol. This goes for surface-active biomolecules, too, and enrichment factors are comparable to those observed for microbes (*e.g.,* Gershey, 1983; Cochran *et al.,* 2016). Compounds that have enriched concentrations in marine aerosols are CHO and CHOS molecular series, and molecules that are more aliphatic (open carbon chain), lower in oxygen content, and smaller. Many of these molecules correspond to homologous series of oxo-, hydroxyl-, methoxy-branched fatty acids, and mono-, di-, and tricarboxylic acids and monoterpenes and sugars. If such materials are present in the ocean of Enceladus, future plume fly-through missions should find them in higher concentrations than in the ocean itself, just as they would find microbes at higher concentrations, with similar EFs.

Compounds such as CO_2_ and CH_4_ have been measured in the plume vapor by the Ion and Neutral Mass Spectrometer at ∼1% by volume (Waite *et al.,* 2009). More recently, higher-mass, more complex organic compounds have been detected (though not fully identified) in E ring ice particles by CDA, at the level of a “few percent” (Postberg *et al.,* 2017). These particles, of course, come from Enceladus and must be present in the plume as well. A concentration at this level would yield, in a single transect of the average plume at 50 km, ∼1 nL of organic material on a 0.04 m^2^ collecting plate. Though meager, these amounts are easily detectable by present-day, post-Cassini mass spectrometers (Lunine *et al.,* 2015) and microfluidics instruments that measure chirality (Mathies *et al.,* 2017, in this issue) in a single transect through the plume.

### 6.3. Other considerations

If bubble scrubbing is at work on Enceladus, it could increase the number of microbes that could be collected in a single transect by factors of thousands and maybe more, and could well be responsible for our detection of organic compounds in the plume (Postberg *et al.,* 2017). (However, as mentioned above, the specific process described by Postberg *et al.* [2017]—bubbles intercepting an organic film residing at the top of the water table and ejecting those compounds as the bubbles burst—may not be at work on Enceladus if turbulent mixing [Kite and Rubin, 2016] is occurring in the fractures.)

There are also other considerations that improve the prospects for detecting microbes. First, numbers of collected microbes can be increased through mission design. An orbiter of either Saturn or, even better, Enceladus would allow repeated passages through the plume. With 10 flybys, for example, instruments that can collect and store their samples over very long periods can increase 10-fold the number of items of interest in the final sample. If gathering intact organisms—alive or dead—were deemed important, impact speeds need to be low: naked bacteria are destroyed upon impact at speeds ∼2 km/s (Mileikowsky *et al.,* 2000), though they could probably remain intact at somewhat faster speeds if encased in ice. While relative flyby speeds for a Saturn orbiter with a closest approach distance of 50 km would be typically ∼ 2–10 km/s, an Enceladus orbiter could fly through the plume at an even lower altitude—say 10 km—with an orbital speed of only ∼ 170 m/s. This is comparable to the speeds with which jet ice particles, on ballistic trajectories, get ejected from their vents (Section 5); they subsequently fall on the surface with the same speed. At 170 m/s, nondestructive capture would be assured, ice encasement or not. Furthermore, a fly-through of the plume for a 10 km altitude orbit would occur every ∼ 2.8 h. In 12 Earth days, 100 fly-throughs could easily be executed, increasing the bounty by another factor of 10 over a 10-Enceladus-flyby Saturn orbiter, so long as the spacecraft carried sufficient power to execute such a mission. Unfortunately, while a Saturn orbiter is well within reach for a NASA New Frontiers mission (Lunine *et al.,* 2015), an Enceladus orbiter is not.

Of course, a lander, though not possible under any other guise but a NASA Flagship, could collect the largest sample sizes of all at the lowest speeds. We find from our modeling that 68–93% of Enceladus plume particles fall back to the surface, the lower value corresponding to the higher typical ejection velocities, and they hit the surface at speeds far less than fly-through speeds: ∼150 m/s versus ∼5 km/s. A catch-plate to intercept falling particles until a sufficiently hefty sample was collected, and a mechanism for retracting it and distributing the cache to the instrument payload, is all that would be required, and is superior to a typical Mars lander soil collection scenario, that is, with a robotic arm that collects samples of regolith on the surface. The analysis of surface ice of indeterminate age, exposed for an unknown period of time to meteoritic and charged-particle bombardment, annealing during Enceladus' southern winter, and so on—in other words, ice that is exceedingly processed—would require more assumptions and caveats, and be far more model-dependent, than that of the fresh samples caught in free fall.

Kempf *et al.* (2010) calculated the rate at which Enceladus frost would accumulate on the surface under the assumption of a mass ejection rate of 5 kg/s. At this rate, they estimated that the accumulation rate in the vicinity of the geysers on the SPT is ∼0.5 mm/year. For collection over one Enceladus orbit, it is more appropriate to use an average mass production rate of 29 kg/s. The mass accumulation rate, using the density of water, then becomes ∼2.9 kg/yr/m^2^ or 0.33 g/h/m^2^. If our estimates for plume bioload are correct, a collecting plate 0.04 m^2^ on a lander would collect ∼0.4 gm, or 0.4 mL of water and ∼4 × 10^3^ microbes in a single 33 h Enceladus orbit around Saturn—an increase of ∼4000 over what a single 50 km altitude transect would collect. Over 100 Enceladus days (or 138 Earth days), the sample size collected by a lander would be ∼10^5^ cells in a water volume of ∼40 mL. These estimates are now well within the range of current microimaging capabilities (Bedrossian *et al.,* 2017, in this issue). If bubble-scrubbing enhancement is at work, these estimates are hundreds to thousands of times higher. On the surface of Enceladus, where the energetic electron fluxes at 0.1, 1, 10, and 100 MeV are, respectively, 500, 20, 10^5^, and 10^9^ times less than at Europa (Garrett *et al.,* 2003, 2005), these organic and microbial loads would have no trouble remaining viable. It is reasonable to assume that a lander could be operational for long enough on Enceladus to return high signal-to-noise results as well as collect sufficient material to make repeat, corroborative measurements.

## 7. Discussion and Conclusion

We have found that the apparent discrepancies among the particle number densities and/or mass production rates from the different Cassini instruments and data analyses, initially on the order of a factor of 10–20, have been reduced to factors of 2–3 by accounting for the differing times and geometries of the observations and the natural, known variabilities in the plume. The remaining discrepancies could easily be the result of poor assumptions about the shape of the plume, the different size bins used by different instrument teams, and/or random and unpredictable changes in the plume between observations. Hence, there is no need to assume that the particles in the plume are aggregates, as had been suggested by Gao *et al.* (2016), to bring photometric and *in situ* results into alignment.

From comparisons of our analyses of ISS images with the analyses of other Cassini data by other investigators, we favor a *q* ≈ 3 exponent of the (differential) plume particle size distribution at 50 km altitude. The *q* ≈ 4 at 100 km altitude found by the *in situ* instruments may reflect the expected vertical gradient in size distribution, with relatively more smaller ones at higher altitudes. For *q* = 3, we derive an early-mission mass production rate of 29 ± 7 kg/s that significantly differs from that reported by IE11 because the latter authors neglected the inherent change in plume mass over the brief duration of their observations. The diurnal peak optical depth at 50–100 km altitudes ranges from ∼10^−3^ > *τ* > ∼10^−4^ (early to late) over the dozen years of the Cassini mission.

The solid-to-vapor (S/V) ratio in the Enceladus plume is important for understanding the eruption mechanisms underlying the moon's activity. In general, high S/V values imply most of the particles are frozen liquid droplets. To qualify as high, S/V needs to be larger than the ∼0.01–0.05 expected if the jet particles are instead produced by the expansion, adiabatic cooling, and direct condensation of vapor to solid (Schmidt *et al.,* 2008; Ingersoll and Pankine, 2010). As we have shown here, variation and stochasticity make inappropriate a comparison of instantaneous values of S and V acquired at different times and locations.

In other words, our estimated “early-mission” diurnal average of 29 kg/s in solids needs to be compared to an equivalent, early mission, diurnally averaged vapor production rate. None exists at this time, but we estimate this quantity by examining the results reported by Teolis *et al.* (2017, in this issue) for the same early-mission time period. Their derivations of the vapor rate, as a function of mean anomaly, have been obtained thus far by using both adiabatic- and non-adiabatic gas expansion/acceleration models for the jets: the former yields an average of ∼200 ± 20 kg/s (the circles in Fig. 11 of Teolis *et al.,* 2017, in this issue), and the latter yields ∼450 kg/s (the blue symbols in their Fig. 11). Teolis *et al.* (2017) reported no definitive evidence thus far of a strong diurnal variation in the vapor production rate. However, there are no vapor data at periapse where the plume in solids is faintest; that is, a diurnal variation could be present but not observable with their available data set. For these reasons, we take an average of all their results (all their blue solid squares and circles) as the vapor production rate—450 kg/s—and consider it an upper limit. As mentioned earlier, our result of 29 kg/s is likely a lower limit, as it disregards the dependence on altitude of the size distribution, a relationship which implies that more massive particles are being ejected closer to the surface than we see at 50 km altitude. This yields an upper limiting ratio of S/V > 0.06, that is, the minimum S/V is just above the vapor/liquid boundary, suggesting that the particles come from liquid. There are of course other reasons to infer a liquid source for the ice particles, such as their salinity, which only makes sense if they are derived from a salty liquid reservoir.

Obviously, the spectrum of drop sizes in the spray, and therefore to some degree the size distribution of the ice particles, in the Enceladus plume will be determined by the size spectrum of the bubbles that produce the spray. Working backward, we know that the liquid-water-derived particles in Enceladus' plume at 50 km altitude are in the range of ∼1 to 10 μ. (Smaller ones are believed to be condensed vapor.) If these come from jet drops and no other process has worked to significantly increase the original sizes, then the parent bubbles at the time of bursting must be very small, in the range of 10 to 100 μ. Bubbles rising through a column in which pressure is decreasing would grow in size, implying that they formed at even smaller size. Based on this argument, it seems more plausible that the particles are frozen film drops. If so, in the absence of other size-altering process, the parent bubbles are ∼1–3 mm in size.

At 50 km, the inferred size distribution of the plume particles is not peaked as it is in Fig. 9. Of course, the size distribution of the particles at 50 km is not the same as that coming out of the vents: gravity selects against those bigger particles that are propelled upward at smaller velocity. We would expect the size distribution to change with altitude in our images, as has been observed in VIMS data (Hedman *et al.,* 2009), and what we observe at any altitude in the plume could be the sum of both film and jet drop size distributions, plus those very small nanosized particles that might have formed by simple homogeneous nucleation from the vapor, which have a size distribution of their own (*e.g.,* Postberg *et al.,* 2009). Also, it has been suggested that particles that turn to ice immediately upon exiting the top of the water table would collect a mantle of ice as they continued to travel upward, due to the condensation of vapor (Schmidt *et al.,* 2008). As we have no size distribution information at the moment for plume particles as they emerge from the vents, this remains an unsettled issue. However, we note that the size distribution of the plume ice particles, in principle, contains information about the details of the exsolution of volatiles (*e.g.,* their partial pressures) in the upper part of the water column within the fractures, and is itself a valuable constraint, even in the absence of any observations of organics or microbes.

It is clear that a future mission at Enceladus could be very productive in the search for evidence of life and even microbes. Estimates of the moon's ocean bioload, assuming its hydrothermal vent cell concentrations are scaled to those at Lost City by the ratio of its geothermal oceanic energy flux to that of Earth's, indicate Enceladus' hydrothermal vents could have native concentrations of ∼10^5^/mL. If these concentrations are diluted in the plume particles by a factor of 10 (Steel *et al.,* 2017, in this issue), a single transect through the plume at 50 km altitude with a 0.04 m^2^ collector plate would yield, during the plume's “average state,” only ∼1 microorganism. However, if bubble scrubbing is present, it would enhance the presence of trace oceanic biosignatures and microorganisms possibly up to factors of hundreds to thousands; the yield could then be 100–1000 organisms, or greater, in a single transect. The same EFs also apply to noncell organic materials in the ocean. For such surveys to be successful in detecting intact microbes, flyby speeds must be kept below ∼2 km/s. It is not likely, then, that a single-encounter, high-speed sample return mission would be able to return intact molecules or organisms.

However, other mission designs can contribute significant enhancements. Provided instruments are capable of accumulating samples from one transect to the next, the more fly-throughs of the plume, the greater the chances of detecting microbes and the larger the collected sample of biomolecules. If bubble scrubbing is at work, 10 such transects with sample accumulation could net up to 10^3^ to 10^4^ organisms or more. A Saturn-orbiter mission with multiple fly-throughs could be done within the NASA New Frontiers program (Lunine *et al.,* 2015). An Enceladus orbiter could increase the sample size over a single transect by as many fly-throughs, or orbits of Enceladus, as could be executed or for as long as instruments could accumulate samples, whichever is shorter—potentially hundreds of times more than in a single transect. And an Enceladus lander, for which the amount of collected material could be vastly more than any other mission, would net, in a single Enceladus day, 4000 times more microbes than could be obtained in a single transect because of the continuous “snowfall” onto the surface. In 100 Enceladus days (or ∼138 Earth days), if bubble scrubbing is at work and sample accumulation of long duration is possible, a lander's cache could be as large as ∼10^7^ to 10^8^ organisms. A lander would require a NASA Flagship-class budget over $1 billion.

Table 7 presents a summary of the possible sample sizes from various mission scenarios, with and without bubble scrubbing, that we have estimated in this work. We note that, based on H_2_ concentrations at Lost City, and the assumptions that methanogenesis is the sole metabolic process and 10% of the available geothermal energy on Enceladus drives the hydrothermal process that supports biomass production, Steel *et al.* (2017, in this issue) estimated microbial concentrations to be as high as 10^9^ cells/mL at the vents. This is more optimistic than our estimate by a factor of 10^4^ and probably should be regarded as an upper limit. Whether Enceladus trends more in the optimistic direction is not currently knowable. Only future missions to Enceladus will be able to tell.

**Table 7.** Enceladus Estimated Microbial Concentrations: Seafloor Vents and Plume

**ASSUMPTIONS**:		
Enceladus' available geothermal power^a^:	*E*_EN_ ∼ 10 GW	
Enceladus' SPT seafloor surface area:	*A*_EN_ ∼ 1.3 × 10^11^ m^2^	
Enceladus' average oceanic geothermal flux:	*F*_EN_ ∼ *E*_EN_/*A*_EN_ ∼ 0.1 W/m^2^	
Earth's average oceanic geothermal flux^b^:	*F*_EA_ ∼ 0.1 W/m^2^	
Ratio of average oceanic geothermal fluxes:	*F*_EN_/*F*_EA_ ∼ 1	
Microbial concentration at Lost City HTV^c^:	*C*_EA_ ∼ 10^5^ cells/mL	
Average plume 50 km transect column density:	∼3 μL/m^2^	
Collector plate surface area, 20 cm square:	0.04 m^2^	
Collected volume of water, single transect:	3 μL/m^2^ × 0.04 m^2^≈ 0.1 μL	
**SEAFLOOR VENT CONCENTRATIONS (cells/mL)**		
Cell HTV concentrations, *C*_EN_≈(*F*_EN_**/***F*_EA_) × *C*_EA_^d^:	*C*_EN_≈*C*_EA_≈10^5^	
**PLUME CONCENTRATIONS (cells/mL)**	Without Bubble Scrubbing^e^	With Bubble Scrubbing^e^
Dilution to 10%, seafloor to base of ice shell, and through ice shell to the surface^f^	*PL*_EN_ ≈ 10^4^	10^6^ < *PL*_EN_′ < 10^7^
**COLLECTED PLUME SAMPLE SIZES^g^ (# of CELLS)**	Without Bubble Scrubbing^e^	With Bubble Scrubbing^e^
Single transect (Saturn orbiter; sample return)	1	10^2^ to 10^3^
Multi-flyby Saturn orbiter^h^	10	10^3^ to 10^4^
Enceladus orbiter^i^	>10^2^	>10^4^ to 10^5^
Lander (within/near a geysering fracture)^j^	>10^5^	>10^7^ to 10^8^

^a^ Lainey *et al.* (2017), Tobie *et al.* (2017).

^b^ Sclater *et al.* (1980).

^c^ Brazleton *et al.* (2006), Perner *et al.* (2010), Reveillaud *et al.* (2016); HTV = hydrothermal vent.

^d^ Assumes partitioning of geothermal energy and conversion into biomass is identical on Earth and Enceladus.

^e^ Cell concentrations and sample sizes are 10^4^ times larger with more optimistic assumptions (see text; Steel *et al.,* 2017).

^f^ Steel *et al.* (2017).

^g^ Averaged over 13 years' variation in plume state for *q* = 3 model (*i.e.,* ∼ 3 μL in particles of all sizes), or ∼0.1 μL sample collected with 100% efficiency on 0.04 m^2^.

^h^ Assuming sample is accumulated over consecutive flybys, ∼60 Earth days apart, for a total of 10 flybys or ∼600 Earth days. Typical example is ELF. Lunine *et al.* (2015).

^i^ Assuming sample accumulation over at least 100 orbits around Enceladus (∼280 h or ∼12 Earth days).

^j^ Assuming sample accumulation over at least 100 Enceladus days or ∼138 Earth days.

If microbes are present, we might predict some of their properties. They would certainly be chemoautotrophs: no sunlight makes it down through the ice, and certainly not to the bottom of a ≥30 km deep ocean, so photosynthesis is impossible. Judging from the inferred ocean chemistry (Waite *et al.,* 2017), it is possible that methanogens are present. And while there is no evidence for, or against, oxygen or sulfur compounds in the plume, it is not implausible that oxidizing or sulfur-reducing chemoautotrophs are present, too. If the ability to photosynthesize conveys a unique cell wall chemistry, we would not expect enceladan organisms to have it. Also, it is not likely that enceladan organisms are pigmented. In the visible portion of the spectrum, Enceladus is the whitest body in the Solar System, and that goes for its SPT (Porco *et al.,* 2006). It is true that in false-color images showing the UV/IR ratio, the tiger stripe fractures are prominently different than the rest of the SPT, but this coloration is believed to be due to different grain sizes (Porco *et al.,* 2006) and not inherent compositional differences between particles landing close to the vents and those landing elsewhere. Furthermore, if colored materials of any kind were falling on the SPT, it would be noticeable in the regional color of the SPT which, instead, is white.

For a reconnaissance survey seeking to detect microbes, a microimager with a reasonable field of view would be required. Currently, such imagers have spatial resolutions of ∼0.5 μ (Bedrossian *et al.,* 2017, in this issue). Consequently, the smallest organisms we could expect to see, assuming they are there at all, would be the size of the smallest terrestrial prokaryotes but not as small as viruses (tens to hundreds of nanometers, *i.e.,* < 1 μ). This means that the particles that contain them will need to have *r* > 0.5 μ.

The widespread hydrophobicity among microorganisms is a major factor in their efficient attachment to bubbles. And it is clear that surfactant compounds that are biosignatures and desirable to detect and measure on Enceladus become readily attached to bubbles; such compounds are produced by a large number of bacteria and archaea. Chemical compositions of these materials include glycolipids, lipopeptides, lipoproteins, and fatty acids, among others (Desai and Banat, 1997). Marine bacteria produce more surfactant than ground-based strains. Surfactants not only play roles in bubble scrubbing as described here but in ice nucleation as well (Christner *et al.,* 2008). Further studies on EFs of icy world analog organisms could guide estimates of expected values on Enceladus.

It is reasonable to ask whether bubble scrubbing in the water rising up through the fractures in the ice shell and erupting out of the surface might eventually so deplete the column of microorganisms that the process becomes unproductive. Kite and Rubin (2016) investigated the tidally pumped outflowing/refilling of the fractures over a diurnal compressional/tensional cycle, plausibly explaining the power output from the fractures by turbulent dissipation in the fractures, as well as the observed phase lag between the plume's brightness and peak tidal stresses (Nimmo *et al.,* 2014). Their mechanism can be sustained indefinitely—with water kept unfrozen and fractures kept open—when fracture widths are of order 1 m. A similar calculation was done by Nimmo *et al.* (2014). Thus, we can expect the material reaching the surface to be continually mixed and replenished of organisms and organic matter.

If bubble scrubbing is at work, its presence for ocean studies could be a double-edged sword. The enhancement of any organic matter in the spray that forms Enceladus' geysers and plume—molecules or organisms—above their concentrations in the bulk ocean would make confident detection of biosignatures, should they be there, all the easier. But the existence of such processes would also make it more challenging to infer the native molecular ratios and microbial concentrations in the ocean.

## Notes

^1^ Contrary to what was claimed by Ingersoll and Ewald (2017), our quadratic fits do not inadvertently remove excess light from under the plume itself, a danger of which we were well aware and avoided in the work of Nimmo *et al.* (2014). Our method yields coefficients of the *x*^2^ term that are 5 orders of magnitude smaller than the linear coefficient (for an image with a width << 10^5^ pixels) and is therefore equivalent to fitting a straight line joining the “flat” portions of the scan on either side of the plume.

^2^ We determined mass per kilometer by applying Eq. 3 to tabulated values of ∫*I*/*F dA* for 9 km thick horizontal slabs centered 50 km above the surface of Enceladus and dividing by 9.

^3^ This sequence contains eight images, all but one taken with the WAC, viewed through the clear filter. The first two have phase angles (*α*) on Enceladus of 174.7° (scattering angles [SA = 5.3°] and mean anomalies [MA] of 10° and 14°). The next three have *α* = 176.4° (SA = 3.6°) and MA = 56°. The sixth has *α* = 177.4° (SA = 2.6°) and MA = 68°. The last two are a narrow-angle image with *α* = 177.6° (SA = 2.4°) and MA = 71° and a wide-angle image with *α* = 177.75° (SA = 2.25°) and MA = 71°.

^4^ We employ the escape velocity from Enceladus, considered as an isolated body. The launch velocity needed to reach Enceladus' Hill sphere is about 13% smaller—209 m/s vs. 239 m/s (Alvarellos *et al.,* 2002; Bierhaus *et al.,* 2012).

^5^ We use a different notation than IE11; what they call *v*_0_, we call *v*_★_, and what they call *v,* we call *v*_0_.

^6^ Ingersoll and Ewald (2017) found that their fits were sensitive to the median of the velocity distribution, *v*_m_, but not to the functional form, so long as the median speed was in the range 143–150 m/s. For an exponential distribution, the fraction of particles launched at speeds > *v*_0_ is exp(−*v*_0_/*v*_★_), so *v*_m_ = ln(2) × *v*_★_; thus median speeds *v*_m_ = 143–150 m/s correspond to *v*_★_ = 206–216 m/s.

## Abbreviations Used

CDA: Cosmic Dust Analyzer
EFs: enhancement factors
FWHM: full-width half-maximum
IE11: Ingersoll and Ewald (2011)
ISS: Imaging Science Subsystem
MA: mean anomaly
NAC: narrow-angle camera
RPWS: Radio and Plasma Wave Science
SA: scattering angle
SPT: south polar terrain
S/V: solid-to-vapor ratio
VIMS: Visual and Infrared Mapping Spectrometer
WAC: wide-angle camera
